# Duplex sequencing identifies genomic features that determine susceptibility to benzo(a)pyrene-induced in vivo mutations

**DOI:** 10.1186/s12864-022-08752-w

**Published:** 2022-07-28

**Authors:** Danielle P. M. LeBlanc, Matthew Meier, Fang Yin Lo, Elizabeth Schmidt, Charles Valentine, Andrew Williams, Jesse J. Salk, Carole L. Yauk, Francesco Marchetti

**Affiliations:** 1 Environmental Health Science and Research Bureau, Health Canada, Ottawa, ON K1A 0K9 Canada; 2 TwinStrand Biosciences, Seattle, WA USA; 3 Department of Biology, University of Ottawa, Ottawa, ON Canada

**Keywords:** Error-corrected sequencing, Genetic toxicology, Mutation spectrum, Trinucleotide mutation signature, MutaMouse, Mutation susceptibility, Benzo(a)pyrene

## Abstract

Exposure to environmental mutagens increases the risk of cancer and genetic disorders. We used Duplex Sequencing (DS), a high-accuracy error-corrected sequencing technology, to analyze mutation induction across twenty 2.4 kb intergenic and genic targets in the bone marrow of MutaMouse males exposed to benzo(a)pyrene (BaP), a widespread environmental pollutant. DS revealed a linear dose-related induction of mutations across all targets with low intra-group variability. Heterochromatic and intergenic regions exhibited the highest mutation frequencies (MF). C:G > A:T transversions at CCA, CCC and GCC trinucleotides were enriched in BaP-exposed mice consistent with the known etiology of BaP mutagenesis. However, GC-content had no effect on mutation susceptibility. A positive correlation was observed between DS and the “gold-standard” transgenic rodent gene mutation assay. Overall, we demonstrate that DS is a promising approach to study in vivo mutagenesis and yields critical insight into the genomic features governing mutation susceptibility, spectrum, and variability across the genome.

**Supplementary Information:**

The online version contains supplementary material available at 10.1186/s12864-022-08752-w.

## Introduction

Mutations in somatic tissues drive the development of cancer. Assessing the mutagenic potential of environmental exposures is essential to evaluate the risks they pose to human health. Genomic features such as chromosomal location, sequence context and transcriptional status can influence mutation susceptibility [1]. However, to elucidate the mechanisms underlying cancer development, a high-resolution understanding of the interplay between exogenous mutagen exposure and genomic features on mutation induction is required. Since most environmental mutagenesis studies rely on experimental manipulations in the laboratory, a critical knowledge gap is contextualizing the relevance of these findings to mutations observed in human cancers. Recent technological advances have enabled the conversion of whole-genome and exome sequencing data obtained from multiple human cancers into mechanism-associated mutational signatures that reflect the processes leading to cancer [2–4]. These computationally derived signatures provide a framework to relate exposure to mutagens to human cancer development. Indeed, recent work shows that many of these mutational signatures can be reproduced by exposing human cell lines [5] or mice [6, 7] to environmental chemicals. Thus, this line of research shows tremendous potential to identify environmental mutagens that contribute to the mutational spectra observed in human cancers and guide regulatory decision-making regarding hazardous carcinogen exposures.

Transgenic rodent (TGR) gene mutation assays are the current “gold-standard” for in vivo mutagenesis assessment and involve measuring mutations in bacterial reporter genes, for example, the *lacZ* or *cII* genes, that have been artificially integrated into the rodent genome [8]. TGR mutation assays are routinely used to generate data to guide regulatory decision-making; however, the reliance on exogenous bacterial genes limits their utility in quantifying relative mutagen susceptibility because they do not accurately reflect the natural variation in sequence context, genomic location, chromatin structure and transcription status observed across the mammalian genome. Additionally, next-generation sequencing (NGS) of the transgene, isolated from manually picked plaques is required to obtain a mutation spectrum [9, 10]. Thus, while the TGR assays have served the regulatory community well, they have important limitations that reduce their ability to resolve important elements of mutagenesis that might better inform human cancer hazards.

NGS technologies have yielded great insights into the complexity of the genomic changes that occur during carcinogenesis [11–14]. However, the technical error rate of standard NGS technologies (~ 1 ×  10^− 3^) is well above the spontaneous mutation frequency (MF) of normal tissues (1 × 10^− 7^ to 1 × 10^− 8^) [15–17], which makes it difficult to distinguish true somatic mutations from sequencing artefacts. Emerging technologies are improving the accuracy of mutation detection by applying consensus-based error correction methodologies [18]. Duplex Sequencing (DS) is one such error-corrected NGS (ecNGS) method that is able to resolve spontaneous and chemically-induced mutations at ultra-low frequencies directly from extracted DNA [17, 19]. DS technology reduces sequencing-derived errors from 1 in 1000 to 1 in 10 million by independently barcoding and building a consensus sequence for both strands of a DNA molecule and reporting only base calls, including mutations, that are complementary on both strands [17]. Thus, DS has the sensitivity and specificity to accurately identify rare mutations that are induced by a mutagenic exposure and can simultaneously generate MF and mutation spectra data.

As marked variability in mutation rate and type has been observed across the genome due to differences in sequence context, recombination rate, replication timing, transcription status, and gene presence [20], it is crucial to study mutagenesis across a broad representative sampling of the genome. Unlike TGR models, DS can measure mutations in any region or target gene of interest, all the way up to the whole genome [21, 22]. In the current study, we broadened our investigations using a DS panel of twenty 2.4 kb targets, for a total target size of 48 kb, scattered across the mouse autosomes and encompassing a diversity of genic and intergenic regions. We used this panel to measure dose-dependent benzo(a)pyrene (BaP) induced MF and changes in mutational spectra in the bone marrow (BM) of MutaMouse TGR animals. BaP is a class 1 carcinogen and potent chemical mutagen that has been extensively studied *in vivo* [23, 24]. Metabolism of BaP results in the formation of DNA adducts that, if left unrepaired, can lead to mutations [25]. We selected BM for this study due to its common use in mutagenicity testing for regulatory purposes and because it is a known target tissue for BaP carcinogenesis [26].

The objectives of the present study were to: (1) apply DS to investigate the effect of sequence context, transcriptional status, and chromatin state on mutation induction across the genome following BaP exposure; (2) explore the utility of mutation spectra data derived from DS to identify specific mutation types that drive the BaP mutation signature; (3) determine if the identified spectra are consistent with any human cancer signatures; and (4) compare the BaP-induced MF measured using DS to that using the TGR “gold-standard” viral plaque assay in the same animals to evaluate the potential and added value for DS to replace current conventional regulatory mutagenicity tests.

## Materials and methods

### Animal exposure and tissue collection

All animal exposures, handling and methods were approved by the Health Canada Ottawa Animal Care Committee. The MutaMouse animals used in the present study are a subset of mice from a previous study investigating the effect of sampling times on mutant frequency induced by various chemical mutagens [27]. Mice were maintained under a 12 h light/ 12 h dark photoperiod at room temperature of 21 °C and relative humidity of 50% with access to water and food (Teklad Global 14% Rodent Maintenance Diet) ad libitum throughout the study. Adult MutaMouse males, 9–14 weeks of age at the beginning of the exposure, were randomly assigned to dose groups. Briefly, they were exposed by oral gavage to either 12.5, 25 or 50 mg/kg BaP or olive oil (as the vehicle control, VC), for a period of 28 consecutive days. Twenty-eight days following the final daily administration, BM was collected, flash frozen and stored at − 80 °C.

### DNA extraction and library preparation

For each mouse, BM isolated from the two femurs was collected in separate vials and processed by different DNA isolation protocols at Health Canada based on the downstream mutagenic assay. For the TGR assay, BM DNA was extracted using a phenol/chloroform-based method and mutant frequency was calculated in the *lacZ* assay according to the OECD test guideline 488, as previously described [27]. For DS, DNA was extracted from BM using the Qiagen DNeasy blood and tissue kits as described in the Qiagen user manual (Cat. # 69504, Qiagen, Hilden, Germany). Isolated DNA was shipped on dry ice to TwinStrand where it was prepared for DS as previously described [19]. Briefly, 500 ng of DNA was prepared by ultrasonically shearing to a mean fragment size of ~ 300 bp followed by end-polishing, A-tailing and ligating to Duplex Sequencing Adapters (Mouse Mutagenesis Kit, TwinStrand Biosciences Inc., Seattle WA, USA). After an initial PCR amplification, the 48 kb of target regions were enriched using a pool comprising 120-nucleotide biotinylated oligonucleotides in two tandem captures as previously described.

Prepared libraries were sequenced on the NovaSeq 6000 using an average of ~ 250 million raw reads per sample (Illumina, San Diego CA, USA). Resulting sequence data as demultiplexed FASTQ files were processed through the TwinStrand Biosciences DuplexSeq Mutagenesis App™ (Version 3.11.0) hosted on the DNAnexus platform, which contains bioinformatics processing methods as previously described in detail [19]. Briefly, bioinformatics processing involves extracting Duplex Tags, aligning raw reads, grouping the reads by unique molecular identifiers and strand defining elements, error-correction of the read groups via duplex consensus calling, consensus post-processing, re-alignment, and finally variant calling. Within this pipeline, raw reads were aligned with bwa, then read pairs were grouped based on unique molecular identifiers and strand defining elements. The read pairs within their read pair groups were unmapped and error-corrected. Bases with low quality were masked as “N” for ambiguous base assignment, and duplex consensus reads were created. In order to eliminate biases from double counting bases in overlapping paired-end reads, the read pairs then went through balanced overlap hard clipping. The resulting duplex consensus reads were end-trimmed and interspecies decontamination was performed using Kraken [28], a k-mer-based taxonomic classifier. Variants were called using VardictJava [29] with optimized parameters. Identical mutations that appeared in more than one molecule in the same sample were considered to be derived from a clonal expansion event and therefore were only counted once. The pipeline produces a summary of sequencing quality metrics as well as MF, mutation spectra, trinucleotide frequency and MF per target data (TwinStrand Biosciences Inc., Seattle WA, USA.)

To investigate the reproducibility of DS data, isolated DNA from a subset of animals (*n* = 3/dose) was also processed for library preparation at Health Canada and sent for sequencing on a NovaSeq 6000 to Psomagen (Psomagen, Rockville, MD, USA). Protocols for library preparation, sequencing, and bioinformatics processing were as described for the samples processed at TwinStrand.

### Mouse mutagenesis panel

The mouse mutagenesis panel comprises 20 genomic targets spread across the mouse autosomes (~ 2.4 kb each) with two targets on chromosome 1. The targets are a balanced representation of the entire genome with respect to GC-content, trinucleotide abundances, and coding status (9 genic and 11 intergenic). The selected target regions have no known role in cancer and are unlikely to be significantly influenced by positive or negative selection. Targets were also chosen for optimal performance in hybrid capture and contain no pseudogenes (or genes with related pseudogenes elsewhere in the genome) or repetitive elements that could potentially confound alignment or variant calling. A description of the target selection and their chromosomal locations can be found in Supplementary Table 1.

### DS data interpretation and statistical analysis

Estimated MFs and pairwise comparisons were obtained using the “glm” function in R, as described [27]. Estimated MFs by target were obtained using a generalized linear mixed model (GLMM) with a binomial error distribution performed by the “glmer” function of the “lme4” package [30] in R version 3.6.1. Pairwise comparisons based on dose, transcription status and chromatin state were estimated using an approach described by Soren and Halekoh, using the “doBy” R package [31]. In these analyses, the Wald statistic is used. The *p*-values from the hypothesis tests comparing the MFs at each dose to controls were adjusted for multiple testing using the Holm-Sidak correction. This multiple testing correction was applied within each chromatin state independently. The chromatin state of the mutagenesis targets and the GC-content of the DS mutagenesis panel were obtained for the mm10 reference genome through the UCSC and NCBI genome browsers (http://genome.ucsc.edu/, https://www.ncbi.nlm.nih.gov/). The location of a target in a dense region on the database ideogram was used as a “best-guess” of chromatin status, which varies by cell type. Gene expression levels, quantified as reads per kilobase of transcript per million reads mapped, were inferred from the NCBI gene database (https://www.ncbi.nlm.nih.gov/). As BM data were unavailable, expression levels in spleen cells of adult mice were used as a surrogate. Like the BM, the spleen is a major hematopoietic organ in mice.

To determine which mutation substitution types differed between the control and treated groups, a modified contingency table approach was used as described by Piegorsch and Bailer [32]. The BaP mutational spectra was then compared against the Catalogue of Somatic Mutations In Cancer (COSMIC) Single Base Substitution (SBS) signatures (available at https://cancer.sanger.ac.uk/signatures/) using cosine similarity values calculated in R, as described [6].

To determine whether any subtypes of mutations were more strongly associated with BaP vs. control samples, we first used the “vegdist” function from the “vegan” package [33] in R to calculate the binomial distance between trinucleotide mutation types for each sample. Then, we performed ordination on the resulting dissimilarity matrix using non-metric multidimensional scaling (NMDS) with the “ordinate()” function in the “phyloseq” package [34]. Briefly, this analysis searches for both a non-parametric monotonic relationship between the sample-to-sample dissimilarities and the Euclidean distances between items to find a location of each item in the low-dimensional space. The relationship is found by regressing distances in this initial configuration against the observed distances. The coordinates are determined by minimizing the stress written as:

$$Stress=\sqrt{\frac{\sum_{i=1}^n{\left(f\left({x}_i\right)-{d}_i\right)}^2}{\sum_{i=1}^n{d}_i^2}},$$

Where x denotes the vector proportions for each trinucleotide mutation, f(x) a monotonic transformation of x, and d is the observed distance.

We also used a nearest shrunken centroids (NSC) approach using the “pamr “package [35] to classify samples based on dose of BaP. In this analysis, only the controls and the BaP high dose samples were used. Here, NSC calculates centroids for the controls and BaP high dose and shrinks the centroids toward 0 using soft thresholding. The samples from the BaP low and mid doses were then assigned to the class (control or BaP high dose) with the minimum distance between each observation and the shrunken centroid. The soft threshold or delta of 3.653 was chosen. This threshold minimized the 6-fold cross validation error and was the most parsimonious model. Then, the Gaussian linear discriminate was used to estimate the probabilities of class membership for samples in the BaP low and mid dose groups. The Gaussian linear discriminant used to estimate the probability of class membership is of the form:

$$ProbBaP=\mathit{\exp}\left(-A/2\right)/\left(\mathit{\exp}\left(-A/2\right)+\mathit{\exp}\left(-B/2\right)\right)$$

$$A=\left(\left(X1-4.23\%\right)/1.81\%\right)2+\left(\left(X2-10.3\%\right)/4.11\%\right)2+\left(\left(X3-4.00\%\right)/1.93\%\right)2\ for\ BaP$$

$$B=\left(\left(X1-1.38\%\right)/1.81\%\right)2+\left(\left(X2-1.64\%\right)/4.11\%\right)2+\left(\left(X3-0.80\%\right)/1.93\%\right)2\ for\ non- BaP\ or\ Control$$

Where A and B are the squared distances to the centroids.

To establish whether induced mutations differed between genic and intergenic targets, we also split the trinucleotide mutation data into separate matrices based on location within the genome and performed the distance calculations as described above. Finally, we used the Mantel test (“vegan” package) to compare each of the distance matrices to one another (i.e., all data, intergenic data, genic data) in a pairwise manner.

### DS and *lacZ* comparison

Estimated *lacZ* MFs were determined as described [27]. The *lacZ* MFs were calculated using only a subset of the mice for which we also had DS data. The MFs obtained with DS versus the *lacZ* mutant frequencies were plotted, fit to a regression line and a correlation coefficient was determined in Excel. Finally, benchmark dose (BMD) analyses were conducted using PROAST in R (version 70.1, https://rivm.nl/en/proast). A benchmark response (BMR) of 50% was chosen, as recommended by White et al. [36]. The dose-response data were fit to both the 3- and 5-parameter Hill, Exponential, Inverse Exponential, and Log-Normal models. These models were then weighted equally to determine a model averaged BMD.

## Results

### DS data yield metrics

We used DS to sequence 24 BM samples derived from MutaMouse males exposed to 0 (VC), 12.5, 25, or 50 mg/kg BaP (*n* = 6) by oral gavage for 28 days. Reads were distributed relatively evenly among the 20 genomic targets and across samples (Supp. Fig. 1). Targets were sequenced to an average duplex consensus sequence depth of ~ 14,500x yielding an average of ~ 850 million duplex base pairs per sample. All samples met a minimum target of 500 million duplex bases per sample for a cohort total of ~ 20 billion duplex bases.

### Mutation frequency

**Fig. 1:**
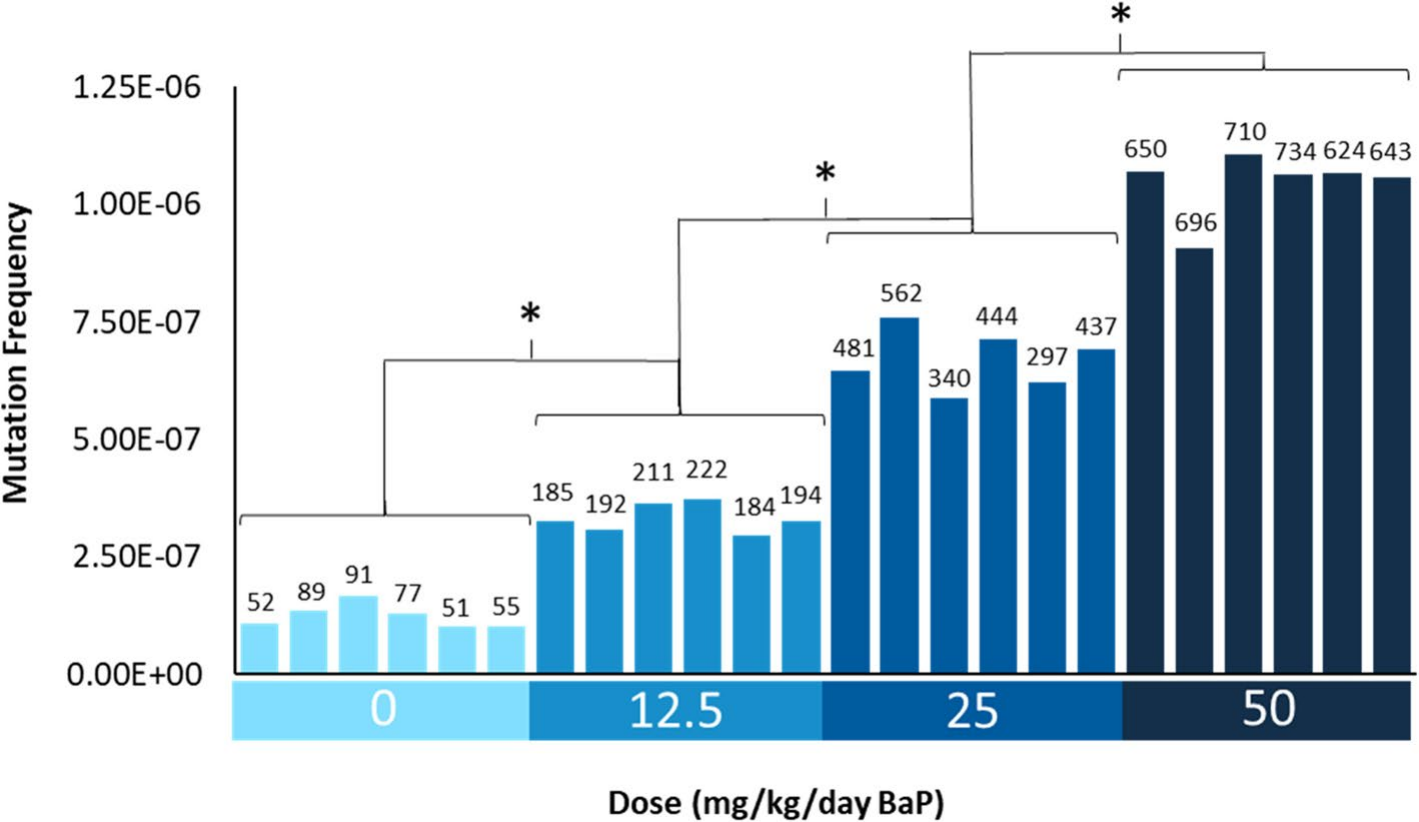
Duplex Sequencing mutation frequencies per bp in the bone marrow of MutaMouse animals by dose. Data are represented as the individual MF for each animal. The number of mutations identified in each sample is shown above each bar. X axis shows the dose groups. * Indicates a significant difference relative to VC (*p* < 0.001) based on pairwise comparisons performed using the doBY package in R with a Holm-Sidak post-hoc analysis

MF was calculated by dividing the number of identified mutant duplex bases by the total number of target-aligned duplex bases sequenced. Mutations that appeared more than once in the same animal were considered to be derived from a clonal expansion event. Thus, only independent somatic mutations contributed to the MF reported. On average, 70, 198, 430, and 676 unique mutations per animal were identified in mice treated with VC, 12.5, 25 and 50 mg/kg BaP, respectively, with minimal intra-individual variability within each dose group (Fig. 1). BaP induced a significant dose-dependent increase in MF relative to VC (*p* < 0.001). Average MFs (× 10^− 7^ ± SD) of 1.3 ± 0.25, 3.3 ± 0.30, 6.8 ± 0.64 and 10.4 ± 0.7 were observed in mice treated with VC, 12.5, 25 and 50 mg/kg BaP, respectively (Fig. 1 & Table 1). BaP induced a 2.7-, 5.4- and 8.4- fold higher MF in 12.5, 25 and 50 mg/kg dose groups relative to the VC mean. An attenuated response was observed with increasing dose of BaP. In fact, BaP induced a mean 2.6-fold, 2-fold and 1.5-fold increase in MF from VC to the low dose, low to the middle dose and middle dose to the high dose, respectively.

**Table 1:** Duplex Sequencing mutation frequencies and *lacZ* assay mutant frequencies in the bone marrow of the same MutaMouse animals exposed to BaP

Assay	Dose (mg/kg/day)	Mean MF^**a**^ (× 10^**− 7**^)	SD (× 10^**− 7**^)	Fold Change^**b**^	Variance (× 10^**− 16**^)	***p*** value
**DS**	0	1.3	0.25		6.1	
	12.5	3.3	0.3	2.5	8.9	2.7 × 10^−9^
	25	6.8	0.64	5.2	41	6.7 × 10^−14^
	50	10.4	0.7	8.0	48	6.7 × 10^−16^
		**(× 10**^**−5**^**)**	**(× 10**^**−5**^**)**		**(× 10**^**−11**^**)**	
***lacZ***	0	4.3	0.6		3.7	
	12.5	109.4	24.3	25.3	5890	1.3 × 10^−13^
	25	313.6	57.4	72.3	32,900	< 1.0 × 10^−16^
	50	572.8	157.1	133	216,000	< 1.3 × 10^− 16^

^a^DS MF shown as mutants per bp sequenced, *lacZ* mutant frequency shown as mutants per locus

^b^Fold change calculated relative to VC

A stronger response was detected when clonally expanded mutations were considered, In fact, estimating clonally expanded mutants by variant allele frequency captured an additional 15, 84, 271 and 1031 mutations; including clonality resulted in average MFs (× 10^− 7^ ± SD) of 1.5 ± 0.40, 4.7 ± 0.65, 10.8 ± 0.69, 26.6 ± 2.8 for VC, 12.5, 25 and 50 mg/kg BaP, respectively.

**Fig. 2:**
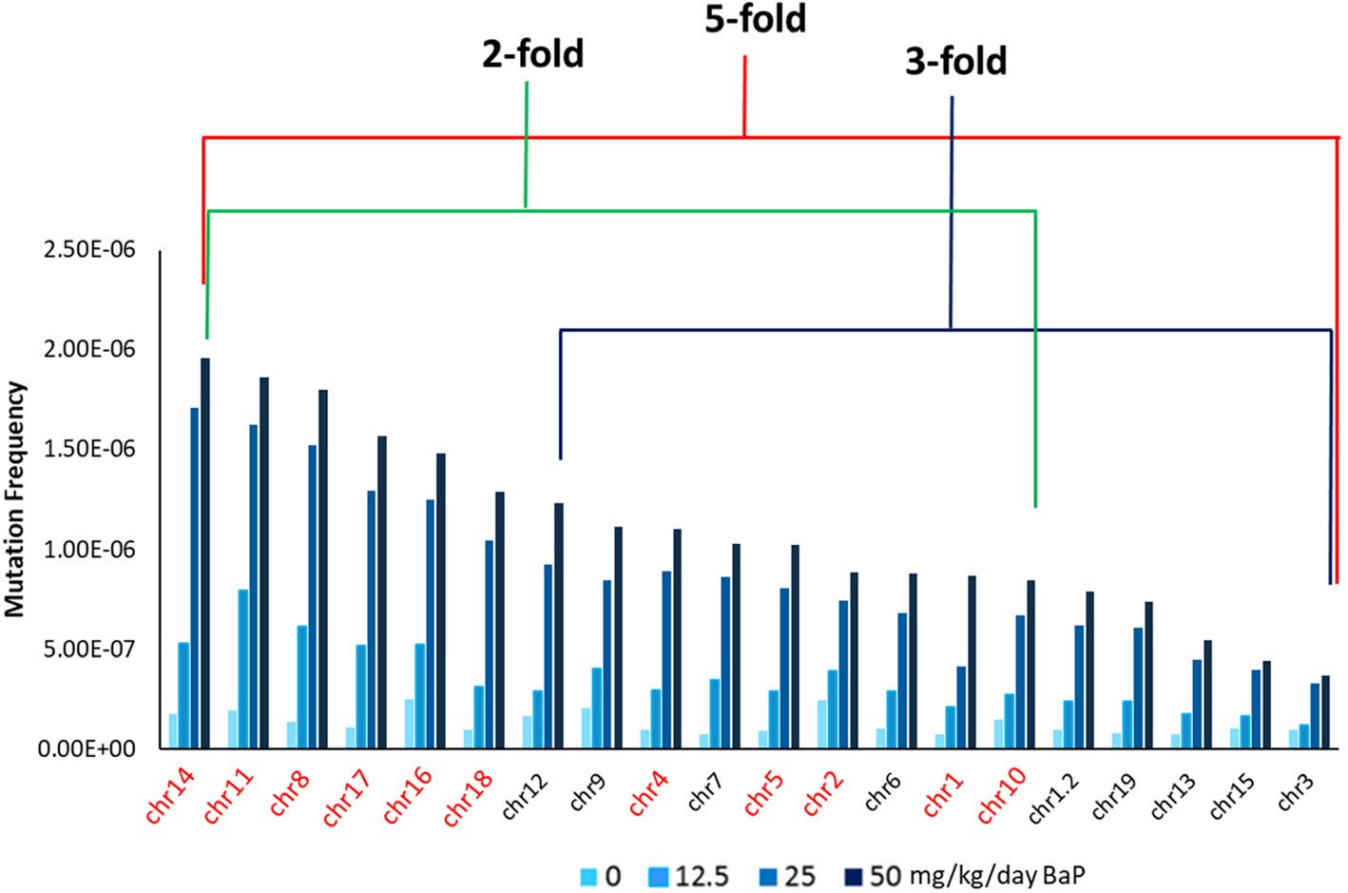
Duplex Sequencing mutation frequencies by the mutagenesis panel target in the bone marrow of MutaMouse animals across BaP dose groups and controls (0 mg/kg/day). Data are represented as the average MF across animals for each individual target. Chromosome number for each DS mutagenesis panel target is shown on the x-axis, with intergenic (red font) and genic (black font) targets specified (chr1.2 denotes the 2nd target on chromosome 1). DS targets are ordered from highest to lowest MF at the high BaP dose (50 mg/kg/day). The red line indicates the fold-difference in MF between the highest MF and the lowest MF at the high dose. The green and blue lines indicate the fold-difference in MF between the highest MF and lowest MF at the high dose within intergenic and genic targets, respectively

Next, we analyzed mutation induction across the 20 individual genomic targets. The individual MF per mutagenesis target are shown in Supplementary Table 2. The background MF (VC) among the 20 targets differed by 4-fold with the highest MF observed at the target on chromosome 16 (1.5 × 10^− 7^) and the lowest MF observed at a target on chromosome 1 (8.6 × 10^− 8^). BaP induced a significant dose-dependent increase in MF across all targets (Fig. 2) with only targets located on chromosomes 3 and 15 failing to show a significant increase in MF in the low dose BaP group with respect to VC. We observed a maximum 5-fold difference between the target with the highest MF at the high and middle dose (chr 14) and the target with the lowest MF at the high and middle dose (chr 3). A higher 7-fold difference was observed between the targets with the highest (chr 11) and lowest MF at the low dose (chr 3). The target located on chromosome 3 had the lowest MF at all BaP doses. Interestingly, targets on chromosomes 11, 14, and 16 were among the five targets with the highest MFs for both BaP (eg, 14, 11, 8, 17, 16) and controls (eg, 2, 16, 9, 11, 14), suggesting that these targets are highly sensitive to both endogenous and exogenous mutagenic factors.

We then analyzed whether mutation induction in the 20 targets was influenced by their location in either a genic or intergenic region. The mean background MF observed in intergenic targets (1.35 × 10^− 7^) was 23% higher (*p* = 0.001) than the mean MF observed in genic targets (1.04 × 10^− 7^). Similarly, BaP-exposed mouse BM had significantly higher mean (across all doses) MF in intergenic (8.8 × 10^− 7^) compared to genic targets (5.1 × 10^− 7^) (*p* < 0.05) (Supp. Table 3).

To further quantify this difference, we determined the percent decrease in mean MF in genic vs. intergenic targets which revealed a 23 and 42% reduction in background and BaP-induced MF, respectively. However, significant differences in MF were also evident within genic and intergenic targets. A 4-fold and 3-fold difference in background MF was observed between the targets with the highest and lowest MF within intergenic (chr 16 and 1) and genic targets (chr 9 and chr 7), respectively. Similarly, a 2-fold and 3-fold difference in BaP-induced MF was observed between the targets with the highest and lowest MF at the high dose within intergenic (chr 14 and chr 10) and genic (chr 12 and chr 3) targets, respectively (Fig. 2).

**Fig. 3:**
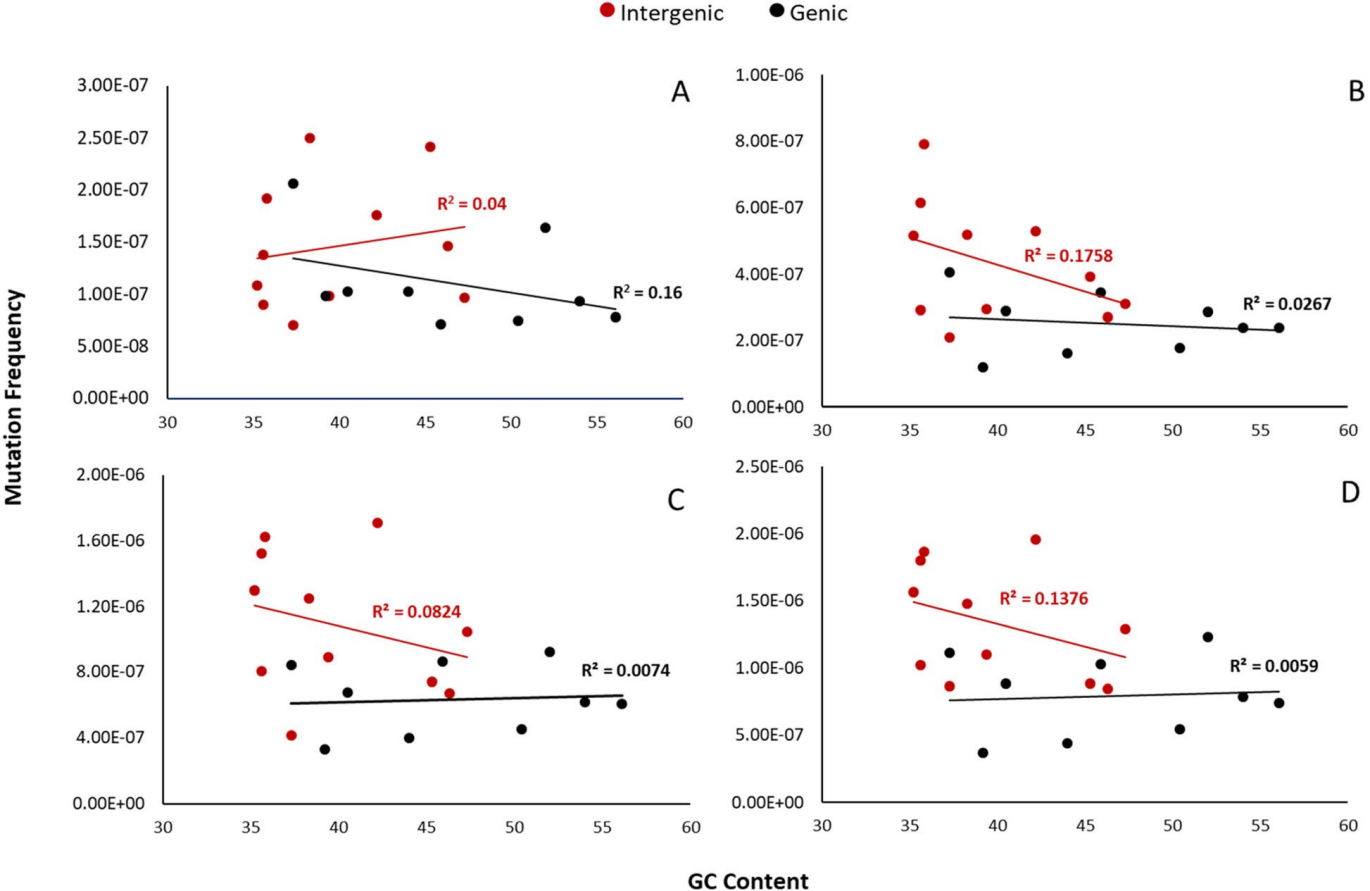
Duplex Sequencing background (**A**) and BaP-induced (**B**-**D**) mutation frequency per target relative to guanine/cytosine content. Data are presented separately for intergenic (red) and genic (black) targets. Vehicle controls (**A**); 12.5 (**B**), 25 (**C**) and 50 (**D**) mg/kg BaP dose

Next, we analyzed whether genomic features such as GC-content, chromatin status, and gene expression levels affected mutation susceptibility. First, as BaP preferentially targets guanine residues, we explored whether variability in GC-content could influence MF. We determined the GC-content of the DS targets that make up the mutagenesis panel (Supp. Fig. 2) and correlated the BaP-induced MF of each target to its GC-content. This analysis did not show a relationship between GC-content and MF in either genic or intergenic targets. In fact, a negative trend was observed in intergenic targets at all BaP doses (Fig. 3). Taking into account only mutations that occurred at a GC-base did not improve the correlation (Supp. Fig. 3). In order to further investigate any interaction between MF and GC-content, we discretized the GC content into two groups based on the mean GC-content (Group 1 < 42.9%, Group 2 > 42.9%) and performed a Type II Wald chi-square test. This additional analysis did not change the outcome (*p* = 0.07).

We then investigated the effect of chromatin state on the MFs observed across targets. Seven of the 20 mutagenesis targets were located within inferred heterochromatic regions. MFs observed in targets located in heterochromatin (1.18 × 10^− 6^ at the high dose) were significantly higher than those observed in euchromatin (9.04 × 10^− 7^ at the high dose) (*p* < 0.001) (Supp. Table 4). Finally, to more precisely investigate whether differences in the MFs observed within genic targets was related to the levels of transcription activity, we used the UCSC database to identify genes contained within the 2.4 kb of each target region and determine their reported expression levels. Since BM data were not available, we used expression levels in the spleen, another hematopoietically active tissue in the adult mouse, as a best guess. A correlation analysis revealed no relationship between expression levels of genes in the genic target regions and their corresponding MF (data not shown). Overall, these results show that MF variation by target was influenced by both genomic location (genic vs. intergenic) and chromatin state, but not GC content or inferred gene expression.

### Mutation spectra

**Fig. 4:**
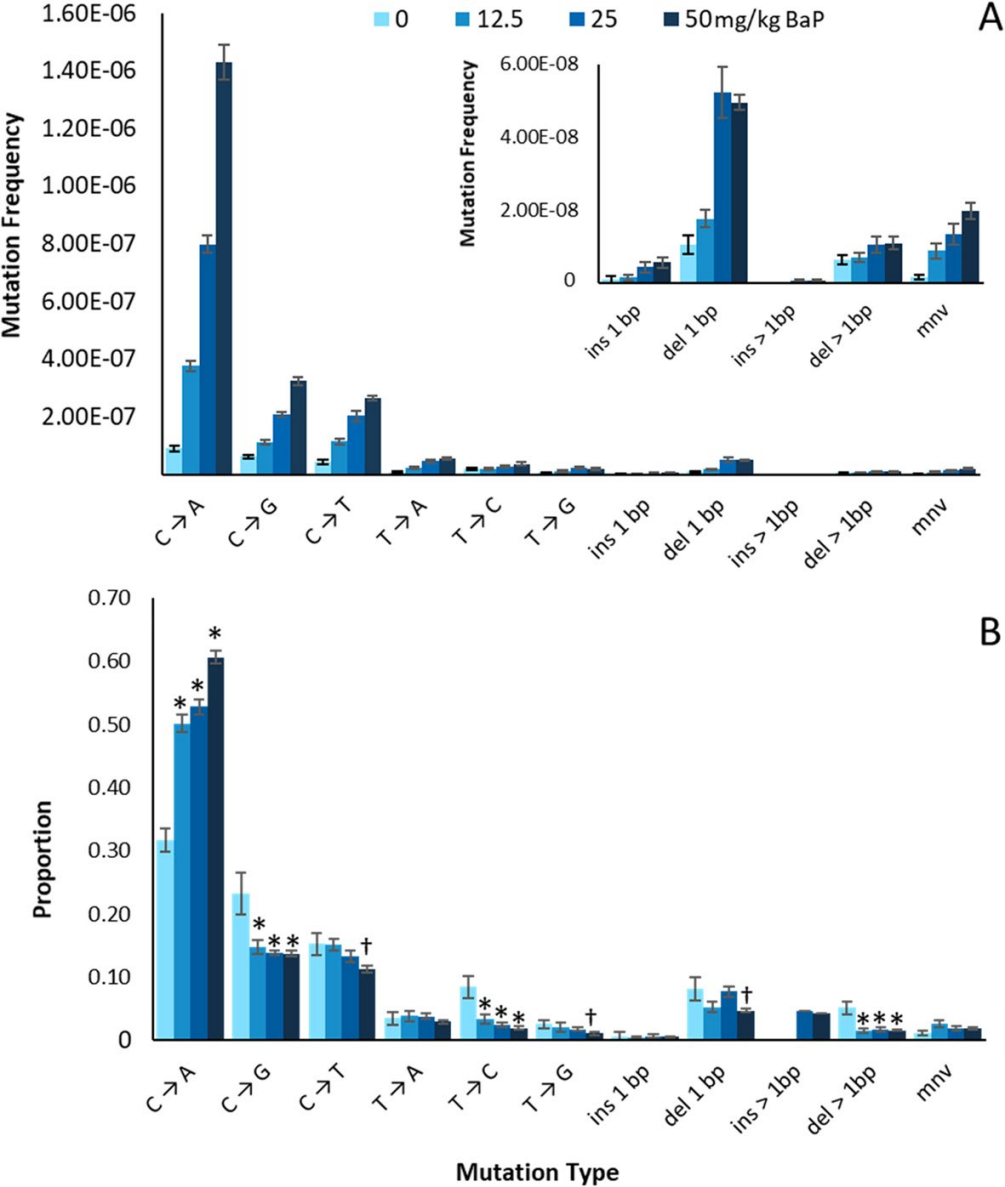
Duplex Sequencing background and BaP induced mutations by subtype in the bone marrow of MutaMouse animals. Mutation subtypes are represented by MFs (**A**) and proportion (**B**). * and † indicate a significant difference relative to VC with *p* < 0.001 and *p* < 0.05, respectively, based on comparisons performed using the doBY package in R with a Holm-Sidak post-hoc analysis. Error bars represent SEM

As expected, our DS analysis revealed that BaP induced primarily single nucleotide variants (SNVs) followed by small insertion and deletion (indel) mutations and multiple nucleotide variants (mnv) (Fig. 4). Pairwise comparison revealed that the overall mutation spectra were significantly different from each other at all BaP doses, largely driven by C > A mutations (*p* < 0.05). Among SNVs, BaP induced primarily C:G > A:T transversion mutations (accounting for 61% of all mutations recovered in the high dose) followed by C:G > G:C transversions (14%) and C:G > T:A transitions (11%). These were also the main background mutations representing 32, 22 and 16% of SNVs among untreated animals. Nevertheless, the overall BaP mutation spectra were significantly different at all doses relative to controls (*p* < 0.001). As BaP dose increased, we observed a significant increase in the proportion of total CpG sites that were mutated (Supp. Fig. 4) reaching 10% at the high dose. No differences were observed in background or BaP-induced mutation spectra between intergenic and genic targets (Supp. Fig. 5).

### Trinucleotide mutation frequencies, COSMIC analyses and classification strategies

**Fig. 5:**
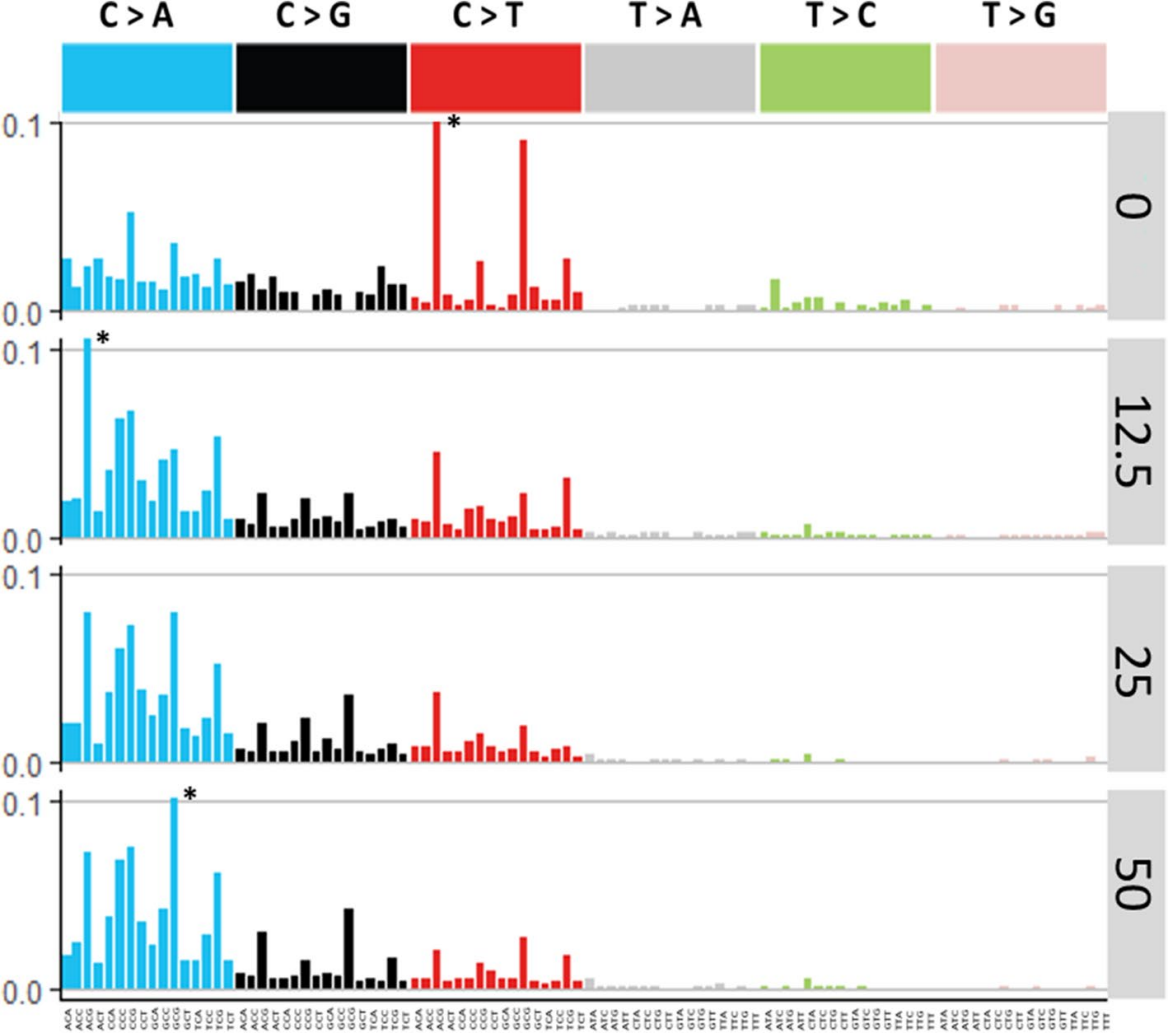
BaP induced trinucleotide frequency context for each BaP dose group and vehicle controls. Data are presented as frequency of each individual mutation type per bp sequenced. * indicates a trinucleotide frequency that is above 0.1 on the y-axis scale

We further analyzed the sequence context of induced mutations by considering the flanking base on either side of the mutated base using two approaches. First, we generated trinucleotide spectra as per the COSMIC approach [4]. When presented in this way, a clear reduction in C:G > T:A mutations and an enrichment in C:G > A:T mutations with increasing doses of BaP became apparent (Fig. 5). We then compared these trinucleotide spectra to SBS signatures of the COSMIC database using cosine similarity. This analysis revealed that the control signature was most similar to SBS 1 (0.73 cosine value), which is an age-associated signature; whereas, the BaP induced mutation spectra was most similar to SBS 4 (0.64 cosine value), which is observed in tobacco-induced lung cancers (Supp. Fig. 6). These results are consistent with expectation given that BaP is a prevalent mutagen in tobacco smoke.

In the second approach, we used exploratory data analysis and classification methods to identify trinucleotides that are most strongly associated with BaP exposure. The ordination of trinucleotide mutation data with NMDS based on the binomial distance revealed a distinct clustering of VC samples with BaP-exposed samples clustering by dose group (Supp. Fig. 7; stress of all data = 0.129; intergenic stress = 0.089; genic stress = 0.116). Typically, a stress less than 0.2 provides a good representation of the data in reduced dimensions, a stress of less than 0.1 provides a great representation. We found that mutation subtypes with a C reference clustered tightly with the BaP-exposed samples, while those with a T reference clustered towards the fringes of the BaP samples. The Mantel statistic, which quantifies the correlation between two distances matrices, revealed that the intergenic and genic subsets had a significant positive correlation suggesting that the higher MF observed in intergenic targets was a result of an increased frequency of the same mutation types rather than differences in mutation spectra (Mantel statistic: 0.7478, significance: 0.001).

Finally, we attempted to create a classifier for BaP/non-BaP samples based on VC and BaP high dose groups using the NSC training model. VC and BaP exposed trinucleotide frequencies were used in the NSC analysis to estimate probability of class membership (VC or BaP-exposed). The standard deviation was used to normalize each trinucleotide so that each one had equal weight when estimating the class membership or the probability of class membership. This analysis showed that 3 mutation types C[C > A]A, C[C > A]C and G[C > A]C correctly classified samples in the low and medium doses as BaP-exposed. Their average frequencies were 1.38, 1.64 and 0.80% in the controls and 4.23, 10.3 and 4.00% in the high dose, respectively (pooled SDs of 1.81, 4.11 and 1.93% for each trinucleotide, respectively).

### DS and *lacZ* assay comparisons

We compared DS MFs to mutant frequencies derived in the same samples using the *lacZ* plaque-based assay and found that there was a strong positive relationship (*R*^*2*^ = 0.94) between the two assays (Suppl. Fig. 8A). However, the magnitude of the response is higher with the *lacZ* assay even when clonally expanded mutations were included. When considering only the intergenic targets, which are not transcribed as the *lacZ* gene, we saw an almost perfect regression line fit (*R*^*2*^ = 0.99). Furthermore, we observed that the DS variance within dose groups is minimal when compared to the *lacZ* assay (Table 1).

BMD modelling is an emerging new approach for establishing point of departure estimates in health risk assessment. BMD analyses yielded BMD values of 1.7 and 6.3 mg/kg BaP for the *lacZ* assay and DS, respectively. The *lacZ* BMD upper and lower bounds of the confidence interval (CI) of 0.4 to 3.3 did not overlap with those of DS at 4.5 to 7.5, indicating a significantly higher BMD for DS. However, when clonally expanded DS mutants were included in the MFs, the resulting BMD CIs of 1.8 and 5.9 surrounding a BMD of 3.7 did overlap with those of the *lacZ* assay suggesting that the results of the two assay are quantitatively similar.

We then compared DS MFs of a subset of animals in this study (*n* = 3/dose) derived from libraries built at TwinStrand and sequenced at Illumina to libraries built at Health Canada and sequenced at Psomagen. As shown in Supplementary Fig. 8B, a strong positive relationship was observed between the two laboratories (*R*^*2*^ = 0.94). An even stronger positive relationship was observed when DS MFs were grouped by dose (*R*^*2*^ = 0.99; data not shown).

**Fig. 6:**
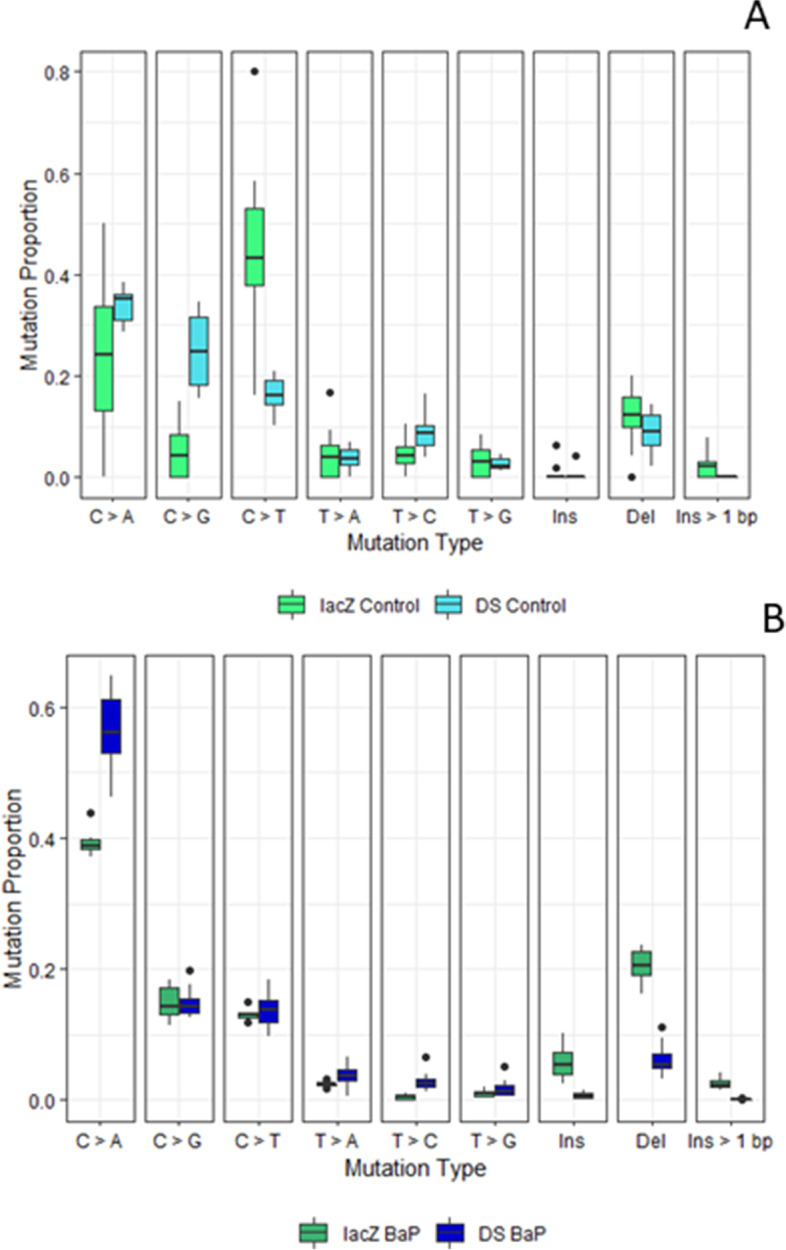
Background (**A**) and BaP induced (**B**) mutation spectra observed in the *lacZ* gene and the Duplex Sequencing mutagenesis panel. Mutations observed in the *lacZ* gene were identified using the TGR mutation assay paired with non-error-corrected NGS [6]. Black dots represent outliers within the data

Finally, we found that the BaP mutation spectra generated by the DS mutagenesis panel was similar to the mutational profile generated by sequencing *lacZ* mutant plaques [9] (Fig. 6B), with C:G > A:T mutations being the most common. More variation in spontaneously induced mutations was observed between assays (Fig. 6A), although this most likely reflected the lower numbers of mutations that were detected in VC samples by both assays.

## Discussion

In this study, we applied DS to investigate background and BaP-induced mutations in the BM of MutaMouse animals across a panel of twenty 2.4 kb targets spanning the genome. As expected, BaP induced a significant dose-dependent increase in MF that was higher in intergenic targets relative to genic targets. Spontaneously-induced MF were also significantly higher in intergenic targets than genic targets, suggesting a protective role of transcription-coupled repair (TCR) for both spontaneous and BaP-induced mutagenesis. Although, BaP induced primarily C:G > A:T mutations, GC-content had no apparent effect on MF across targets. Mutations at C[C > A]A, C[C > A]C and G[C > A]C trinucleotides drove the BaP mutation signature and successfully discriminated exposed animals from VC. Comparison of DS results with those obtained with the OECD approved *lacZ* assay showed a positive linear relationship. Overall, these data suggest that DS enables a comprehensive analysis of in vivo chemical mutagenesis that can provide critical insights into genomic features underlying mutation susceptibility and variability across the genome.

Existing methods for in vivo mutagenesis analyses rely on single bacterial reporter genes or a small region of specific endogenous genes and generally provide a measure of the mutagenicity of the tested compound and little mechanistic information based on mutation type due to the limited size of the selectable locus. The ability to query large regions of the mammalian genome for chemically induced mutations is a major advantage of DS that enabled us to explore mutation susceptibility across a range of loci with different sequence contexts, chromatin states and transcriptional statuses. We observed significant differences in the magnitude of the response across loci and between intergenic and genic targets supporting a role for TCR, a sub-pathway of nucleotide excision repair [37]. Our results in genic targets showing a mean reduction of 23 and 42% for background and BaP-exposed MF, respectively, are consistent with previous observations [38–40]. These results are also consistent with a recent DS study where MF for non-transcribed genes were up to 8-fold higher than transcribed genes [19]. Furthermore, our absolute MF and fold-change with respect to controls are comparable to those reported in the aforementioned study that measured mutations in different endogenous genes and using a different TGR model [19]. These findings show the robustness and accuracy of DS in measuring induced mutations and suggest that when the genomic target queried is sufficiently variable, DS provides a good estimate of the MF across the entire genome.

The use of a panel of 20 targets randomly distributed across the genome enabled an initial analysis of the association between genomic features and susceptibility to mutations. We observed that MF were affected by chromatin status. In fact, the two intergenic targets with the highest MF were found in heterochromatic regions and overall MF were significantly higher in targets located in heterochromatic regions. These regions are densely packed, often located near the centromeres, are typically transcriptionally inert [41] and have been associated with elevated mutation rates in certain cancers [42, 43]. Differing accessibility to DNA repair complexes, variation in the ability to signal repair, or differential exposure to mutagens at the nuclear periphery where heterochromatic regions tend to locate are factors that drive this association. However, we did not observe a correlation between the location of a target in either a genic or intergenic region and its chromatin status, indicating that the relationship between chromosomal organization and mutation rate is complex and requires further study. The ability to investigate such relationships in diverse tissues with customizable DS panels may help with understanding the occurrence of mutagen-induced cacinogenesis in select tissues.

We found no effect of gene expression levels and GC content on measured MF. The negative finding for gene expression may due to the reliance on publicly available spleen gene expression data. Although both BM and spleen are sites of active hematopoisis in the adult mouse, there are small differences in gene expression profiles between the two tissues [44]. Thus, it is possible that obtaining the expression levels in the BM in the animals used in this study could lead to a better correlation between gene expression and mutation susceptibility. The lack of a correlation between GC content and MF is more surprising because BaP preferentially targets guanine residues [9]. In addition, CpG sites are known to be mutational hotspots due to spontaneous deamination of cytosine residues [1]. Interestingly, neither spontaneously derived mutations nor BaP-induced mutations were associated with the GC-content of the targets. It is possible that the higher presence of GC-rich loci in genic regions, which are subjected to TCR, attenuated the higher mutability of GC rich regions [45]. Additionally, the balanced representation of GC-content across targets, which only resulted in a maximum 20% difference between the highest and lowest GC-content, may explain the lack of observed effect. We note that we are underpowered to detect significant differences at the individual target level based on the average number of identified mutations per individual target. Nevertheless, this study highlights the importance of measuring mutagenesis across genomic features to capture the extensive variability in susceptibility.

An inherent advantage of DS over traditional mutation assays is that, in addition to MF, it provides information on the types of induced mutations allowing for the detailed analyses of mutation spectra as a function of BaP dose. As expected, BaP induced mostly C > A transversions that increased in a dose-dependent manner. The observed mutation spectra were consistent between intergenic and genic targets and the known mode of action of BaP, which forms bulky DNA adducts mostly at the N2 of guanine through its metabolite benzo(a)pyrene-7,8-diol-9,10-epoxide [9, 46]. We observed that BaP induced only a small proportion of indel mutations with a majority of them only a single base pair in length (Supp. Table 5). The BaP mutation profile derived in this experiment is consistent with the mutation spectra obtained using different sequencing approaches [6, 19, 47].

We further considered the flanking nucleotides and their contribution to the BaP mutation signature to allow a comparison with COSMIC signatures. The background trinucleotide spectra obtained in this study was highly similar to SBS 1 (cosine similarity [CS] > 0.90). SBS1 is one of the two COSMIC “clock-like” signatures and represents mutations that arise as a function of age [48]. The BaP trinucleotide spectra was most similar to COSMIC SBS 4, SBS 24 and SBS 29 (CS > 0.5 at all doses). The proposed aetiology of SBS4 and SBS 29, tobacco exposure, aligns with the primary route of human exposure to BaP. SBS 24 is associated with aflatoxin exposure; although this is unrelated to BaP exposure it has a close similarity to SBS 4 and SBS 29 (CS of 0.63 and 0.85). This is not the first study in which in vivo chemical exposures have been linked to human cancer signatures [6, 47]. However, the improved accuracy of DS over traditional NGS technologies and efficiencies in mutation characterization provide an advantage in investigating the role of mutagens in the development of human cancers. Overall, we observed that BaP induced a consistent mutation spectrum across doses, coding and non-coding targets, and generated a mutation spectrum that matched closely COSMIC SBS signatures that align with its mutagenic mode of action.

We demonstrate that analyses restricted to single trinucleotides can correctly classify BaP-exposed samples. NMDS analysis revealed distinct clustering of BaP treated samples based on the mutated trinucleotides, while NSC analysis revealed that the three trinucleotide patterns that were most strongly driving the BaP-exposed classification all contained a C:G reference base and two or three repeated adjacent G bases. The NSC classification method identified mechanistically relevant trinucleotide mutations for the mode of action of BaP and, importantly, correctly classified the low and medium dose groups as exposed. Such an approach could be advantageous when there are insufficient reads to produce a robust 96-trinucleotide spectrum that is necessary for the COSMIC analyses. From a regulatory toxicology perspective, we propose that there could be an advantage to the classification approach described here. That is, improving detection sensitivity through characterization of the effects of mutagens on the types of induced mutations rather than basing hazard exclusively on MF.

An additional objective of our study was to compare the performance of DS in assessing in vivo mutations against the gold standard TGR assay. We found that DS and the *lacZ* assay results were strongly correlated (*R*^*2*^ = 0.94); however, the *lacZ* assay showed higher fold increases. An attenuated response was also observed in another study that compared BaP induced mutations by DS relative to the BigBlue® plaque-based assay [19]. These authors proposed this to be a result of unrepaired DNA adducts that were fixed into mutations during the in vitro assay. This is not an explanation for the higher response in the *lacZ* assay observed in this study as it is unlikely that DNA adducts would remain 28 days after the end of treatment. Additionally, Monroe and Skopek et al., observed in two comparative studies in BigBlue® mice that the exogenous *lacI* gene responded to BaP mutagenesis in splenic T cells at a higher magnitude than the endogenous *hprt* gene [49, 50]. Interestingly, significantly more mutations in the *cII* gene versus endogenous genes were also reported in the BM of BigBlue® mice exposed to BaP [19]. Thus, it appears that exogenous bacterial genes may represent a preferential target for BaP mutagenesis, most likely due to an enrichment of motifs that are highly mutable for BaP, specifically, the NCG motif where N is any nucleotide [6]. Indeed, in the current study, the highest mutated trinucleotide motif for the BaP-exposed groups was ACG at the 12.5 mg/kg dose and GCG at the 25 and 50 mg/kg doses. The enrichment of these motifs that are “attractive” to BaP likely contributes to this preferential targeting. In future studies, it would be beneficial to sequence the *lacZ* gene concurrently with the DS mutagenesis panel and sequence at a higher depth to directly compare its response versus endogenous DNA.

There are fundamental differences between the two assays that are also likely to underlie the observed difference in fold increases. First, the *lacZ* gene does not undergo transcription in the MutaMouse and therefore is not subject to TCR. Unlike DS, the calculated *lacZ* mutant frequency does not distinguish between unique mutations and those resulting from clonal expansion events. Clonally expanded mutations can only be identified when the assay is paired with NGS and may artificially and significantly inflate the observed MF [8, 9]. Therefore, we suggest that the lack of TCR in the exogenous bacterial targets and the inclusion of clonally expanded mutations leads to an increase in the mutant frequency calculated for the *lacZ* assay relative to MF analysis of endogenous loci by DS presented herein. Indeed, inclusion of clonally expanded mutants, while not improving the relationship between DS MFs and the *lacZ* assay mutant frequencies (*R*^*2*^ = 0.88), resulted in overlapping BMD CIs between the two assays. Thus, when clonality is taken into account, the BMD response measured by DS is in line with that measured by the *lacZ* assay. This result suggests that when DS is applied to measure mutagenic responses for hazard identification, clonally expanded mutations should be included when evaluating the results.

In summary, this study highlights the strong potential of DS to elevate and transform in vivo mutagenesis assessment. Unlike the *lacZ* assay, the DS panel can be custom designed to include any region of interest. This can allow for panel customization to capture highly mutable or less mutable sites depending on target tissue and mutagen exposure while still capturing endogenous genomic features present across the genome. This is particularly useful when considering carcinogens that may operate only on specific areas of the genome resulting in regional mutagenic effects. Additionally, the inter-laboratory validation performed in this study indicates that DS provides consistent results across laboratories and sequencing facilities. Further work is required to understand the performance of DS with weak mutagens, mutagens with differing modes of action or mutagens acting on tissues with a lower cell turnover than BM. From a health perspective, DS yields novel insights into the genomic features that influence mutation induction, the exogenous exposures that inflict DNA damage, and consequently helps to elucidate potential mechanisms that underlie the development of human cancer. Furthermore, detailed spectra obtained from DS provides the opportunity to classify mutagen exposures based on the specific trinucleotide mutations induced. From a regulatory perspective, DS overcomes many of the limitations associated with conventional mutagenesis assays and can accurately and efficiently provide MF data to be used to inform sound regulatory decision making.

## Supplementary Information

**Additional file 1.**

## Data Availability

Sequencing data have been deposited with the Sequence Read Archive (SRA) under SRA accession number PRJNA803048. Sequencing data processing and bioinformatics analyses were performed on the DNAnexus platform using the TwinStrand Biosciences DuplexSeq Mutagenesis App (Version 3.11.0; https://platform.dnanexus.com/app/twinstrandbio-mutagenesis). Per-target mutation frequency calculation was performed using an R script (Supplementary materials). The workflow used to do NMDS and NSC analyses are available on Github (https://github.com/EHSRB-BSRSE-Bioinformatics/2022_LeBlanc_et_al_BaP_DupSeq).
