# Supplementary material for: Duplex sequencing identifies genomic features that determine susceptibility to benzo(a)pyrene-induced in vivo mutations
Source: BMC Genomics. 2022 Jul 28;23:542. doi: 10.1186/s12864-022-08752-w (PMC9331077; doi:10.1186/s12864-022-08752-w)
Supplement: Supplementary file 1 — Additional file 1. [file 12864_2022_8752_MOESM1_ESM.docx]

**Supplementary Material**

Duplex Sequencing identifies genomic features that determine susceptibility to benzo(a)pyrene-induced *in vivo* mutations

Danielle P.M. LeBlanc^1^, Matthew Meier^1^, Fang Yin Lo^2^, Elizabeth Schmidt^2^, Charles Valentine III^2^, Andrew Williams^1^, Jesse J. Salk^2^, Carole L. Yauk^1,3^, Francesco Marchetti^1,4^

^1^Environmental Health Science and Research Bureau, Health Canada, Ottawa, ON, Canada; ^2^TwinStrand Biosciences, Seattle, WA, USA

^3^Department of Biology, University of Ottawa, Ottawa, ON, Canada

**Supplementary Table 1. Mutagenesis panel target characteristics**

**
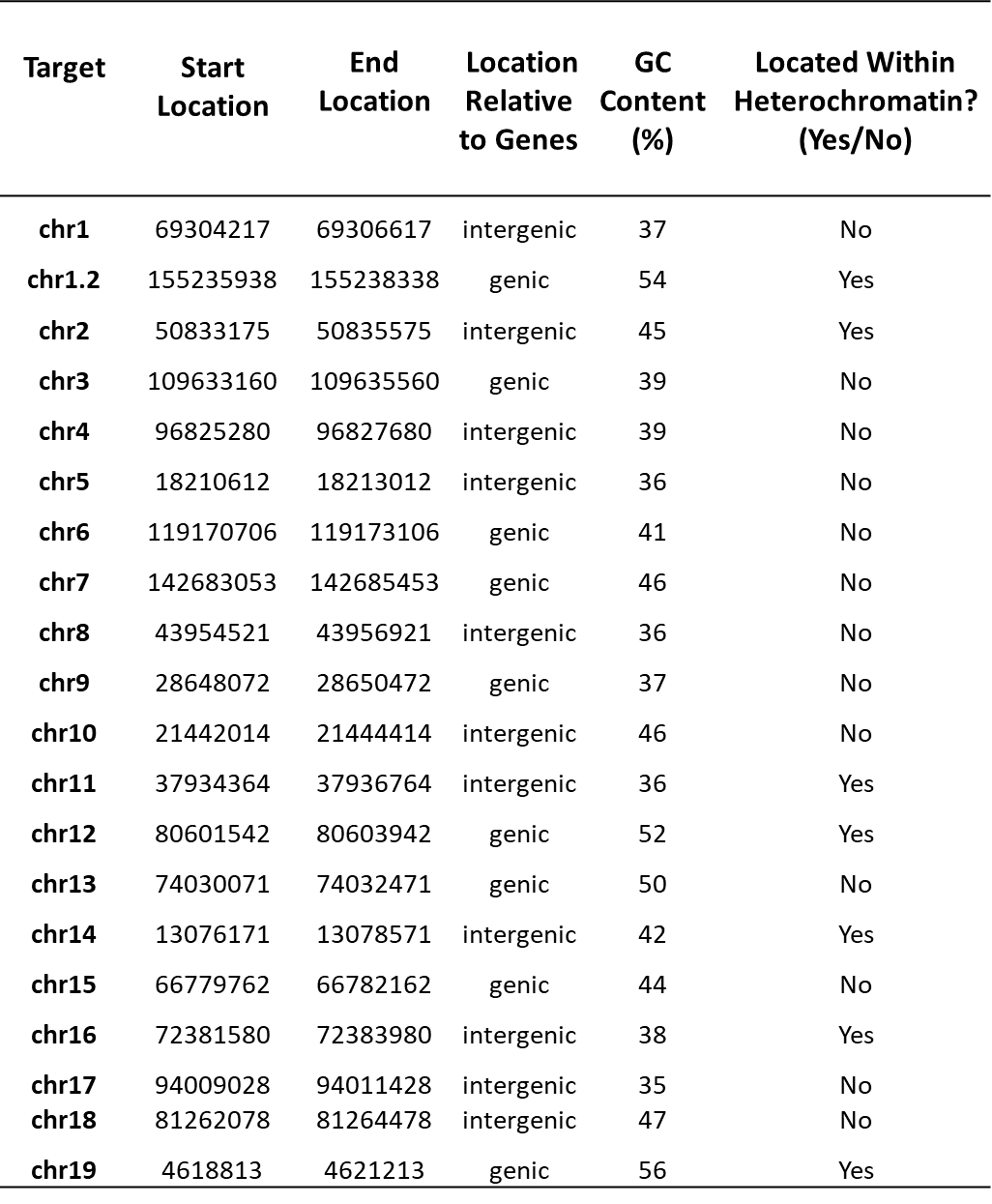
**

The TwinStrand Duplex Sequencing Mouse Mutagenesis (Mouse-50, v1.0) hybrid selection panel describes twenty 2.4kb genome-representative targets within the mm10 genome assembly. This panel was designed to quantify and characterize background and induced mutations in the autosomal genome of the mouse while controlling for biases due to sampling a small portion of the overall genome. To ensure high on-target capture efficiency and overall sequencing quality, the panel was built to avoid repetitive elements as defined by UCSC mm10 RepBase. An additional technical optimization of the panel ensures that the targets have near-perfect mappability scores and no homopolymers of length 8 or more^1^.

All baits within the target regions are also optimized such that they, and the genomic fragments they will pull down, uniquely map to the reference genome and have at least a predicted minimum free energy of -15 as determined by ViennaRNA configured under a DNA folding mode^2,3^.Targets are uniformly spread across the autosome such that almost all autosomes have at least one target except for chromosome 20 which did not have any qualifying targets. In addition, all targets contain a balanced overlap of genic, inter-genic, and non-genic regions that is similar to the abundance of coding and non-coding regions genome-wide. Finally, every target was selected such that it has no known role in mouse cancer or in a genomic region that has a homologous relationship to cancer in the human species.

1. Karimzadeh, M., Ernst, C., Kundaje, A., Hoffman, M.M. (2018) Umap and Bismap: quantifying genome and methylome mappability. *Nucleic Acids Research.,* 46, e120.
2. Lorenz, R., Bernhart, S.H., Höner zu Siederdissen, C., Tafer, H., Flamm, C., Stadler, P.F., Hofacker, I.L. (2011) ViennaRNA Package 2.0. Algorithms for Molecular Biology., 52, 26.
3. Turner, D.H., Mathews, D.H. (2010) NNDB: the nearest neighbor parameter database for predicting stability of nucleic acid secondary structure. Nucleic Acids Res., 38,280–282.

**Supplementary Table 2. Mean mutation frequency estimates and mutation and background counts per target per dose.**

**
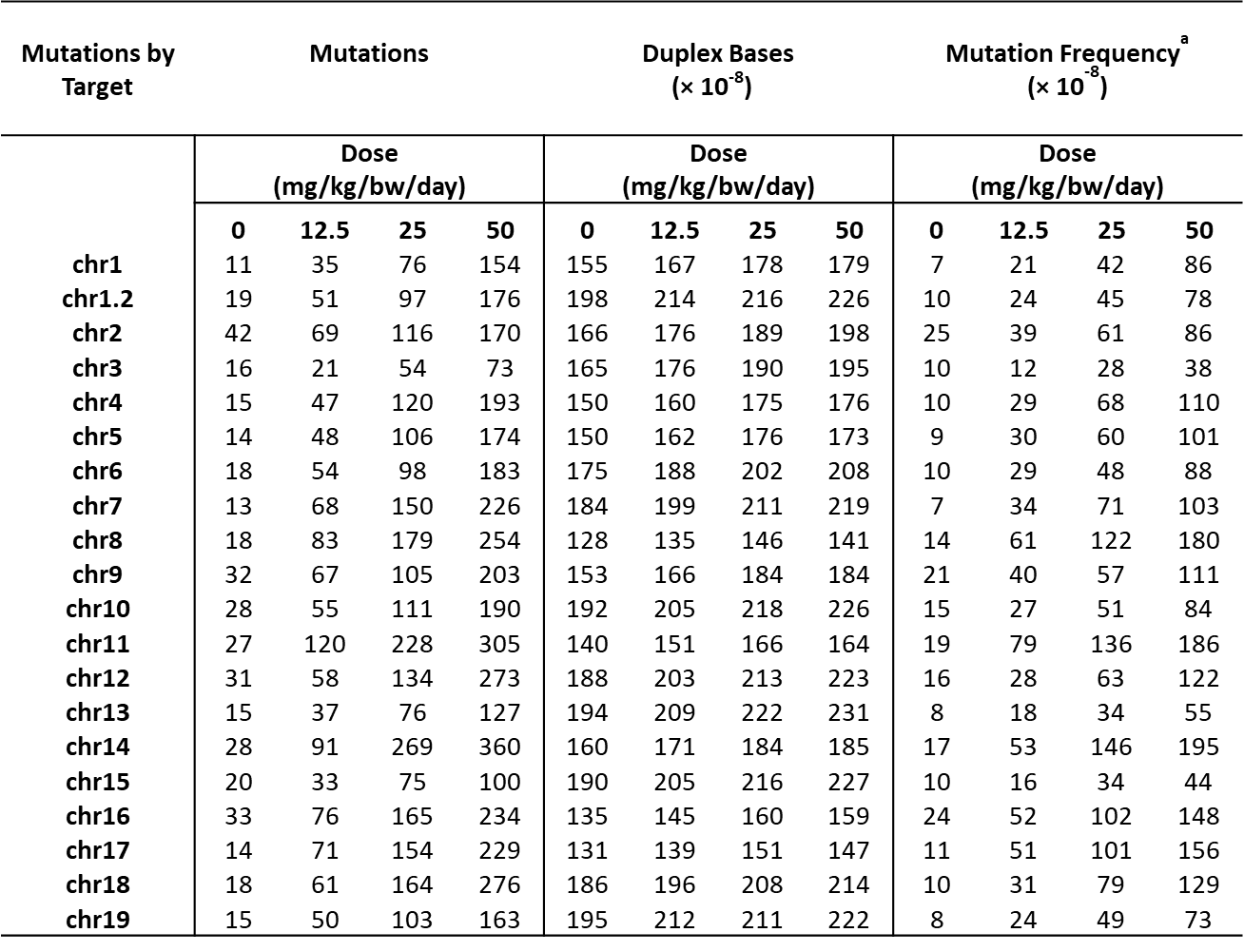
**

^a^DS MF shown as mutants per bp sequenced


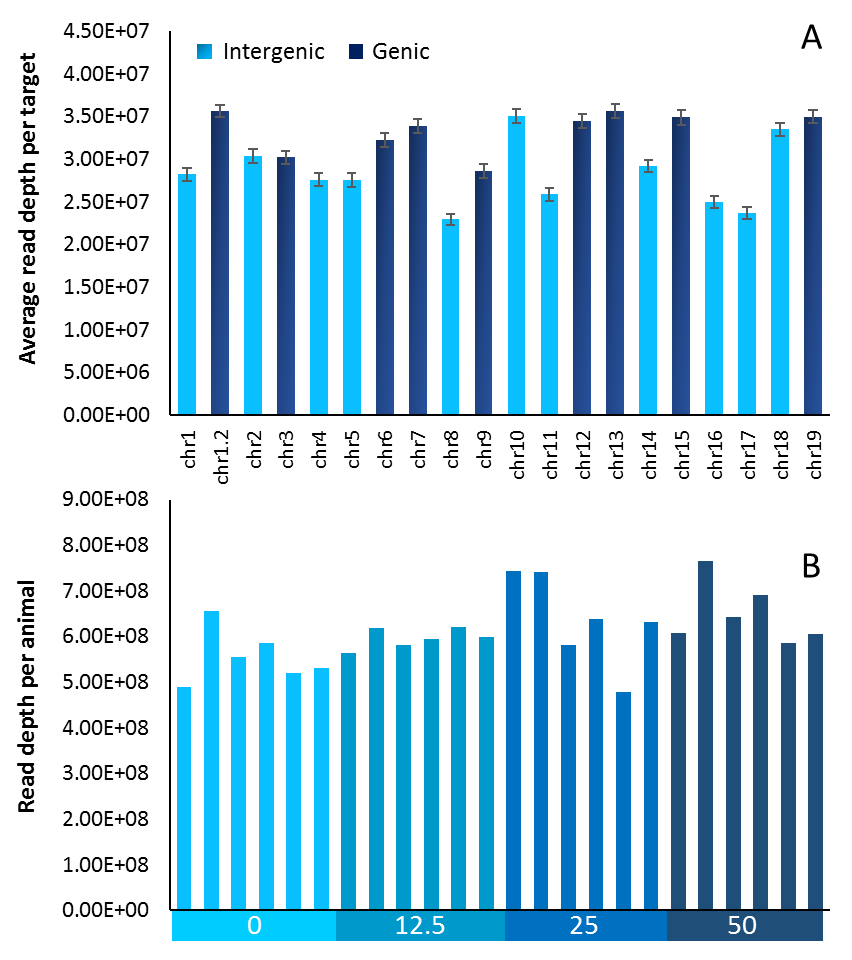


Supplementary Figure 1. Duplex Sequencing coverage for each target in the mutagenesis panel and by MutaMouse animal.

(A) DS average read depth per target by chromosome location. Error bars represent standard error of the mean (SEM). (B) DS average read depth per individual animal. X-axis indicates dose group (mg/kg/day BaP).

**Supplementary Table 3. Mean mutation frequency estimates in intergenic and genic targets and corresponding pairwise comparisons**

**
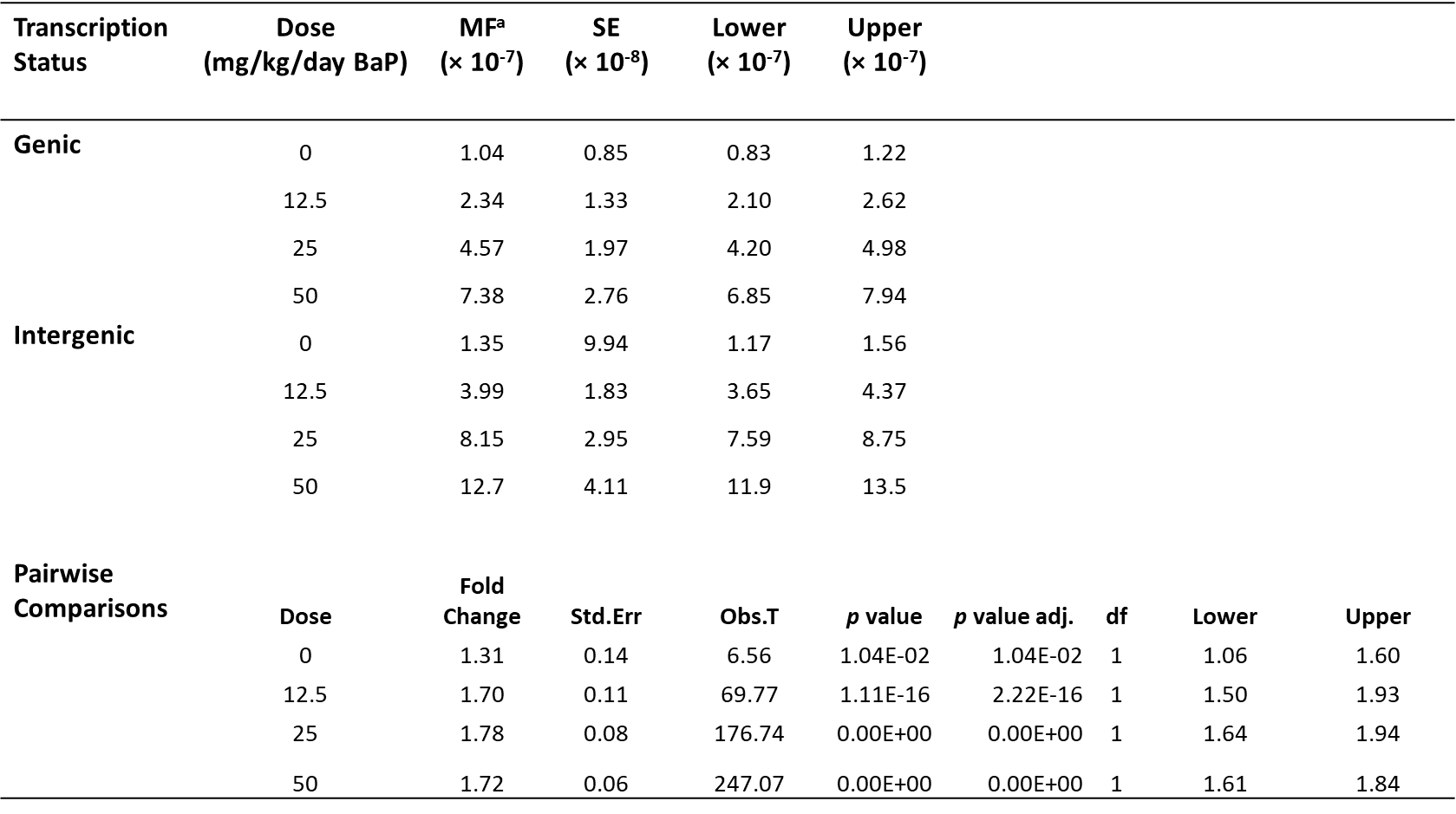
**

^a^DS MF shown as mutants per bp sequenced

**
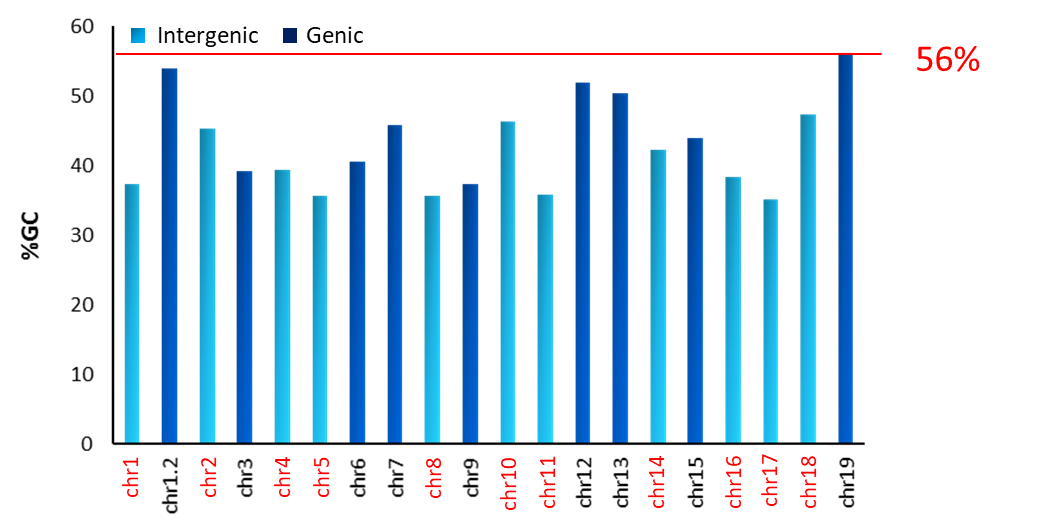
**

Supplementary Figure 2. Percent GC-content of Duplex Sequencing mutagenesis targets.

Data is represented for both intergenic (light blue) and genic (dark blue) targets. A red line with associated number indicates the GC-content of the *lacZ* gene.

**
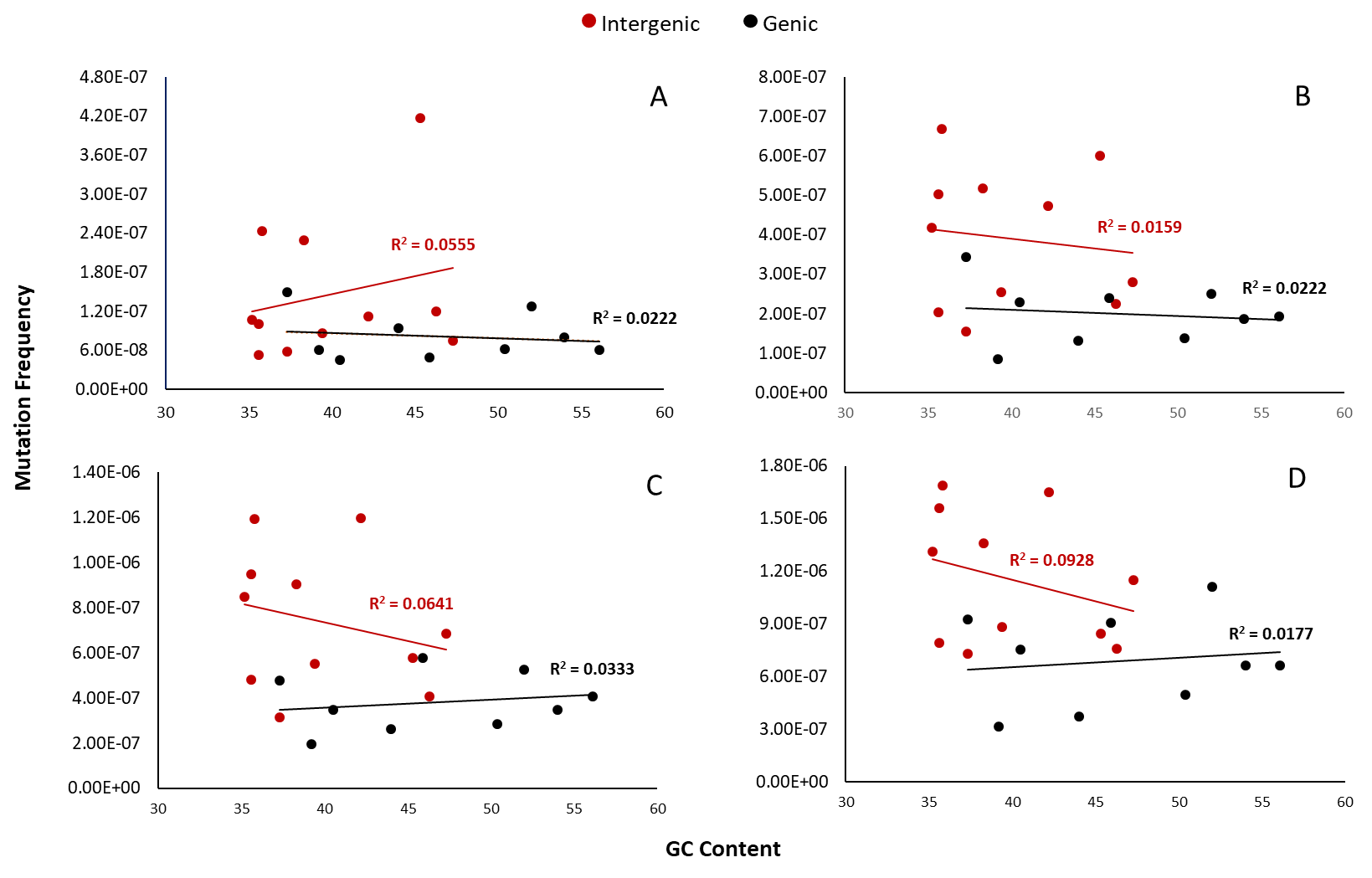
**

**Supplementary Figure 3. Duplex Sequencing background (A) and BaP induced mutation frequency per target, taking into account only mutations that occurred at a C or G base, relative to guanine/cytosine content.**

Data are presented separately for intergenic (red) and genic (black) targets. Vehicle controls (A); 12.5 (B), 25 (C) and 50 (D) mg/kg BaP dose.

**Supplementary Table 4. Mean mutation frequency estimates by chromatin state of the mutagenic targets and corresponding pairwise comparisons**

**
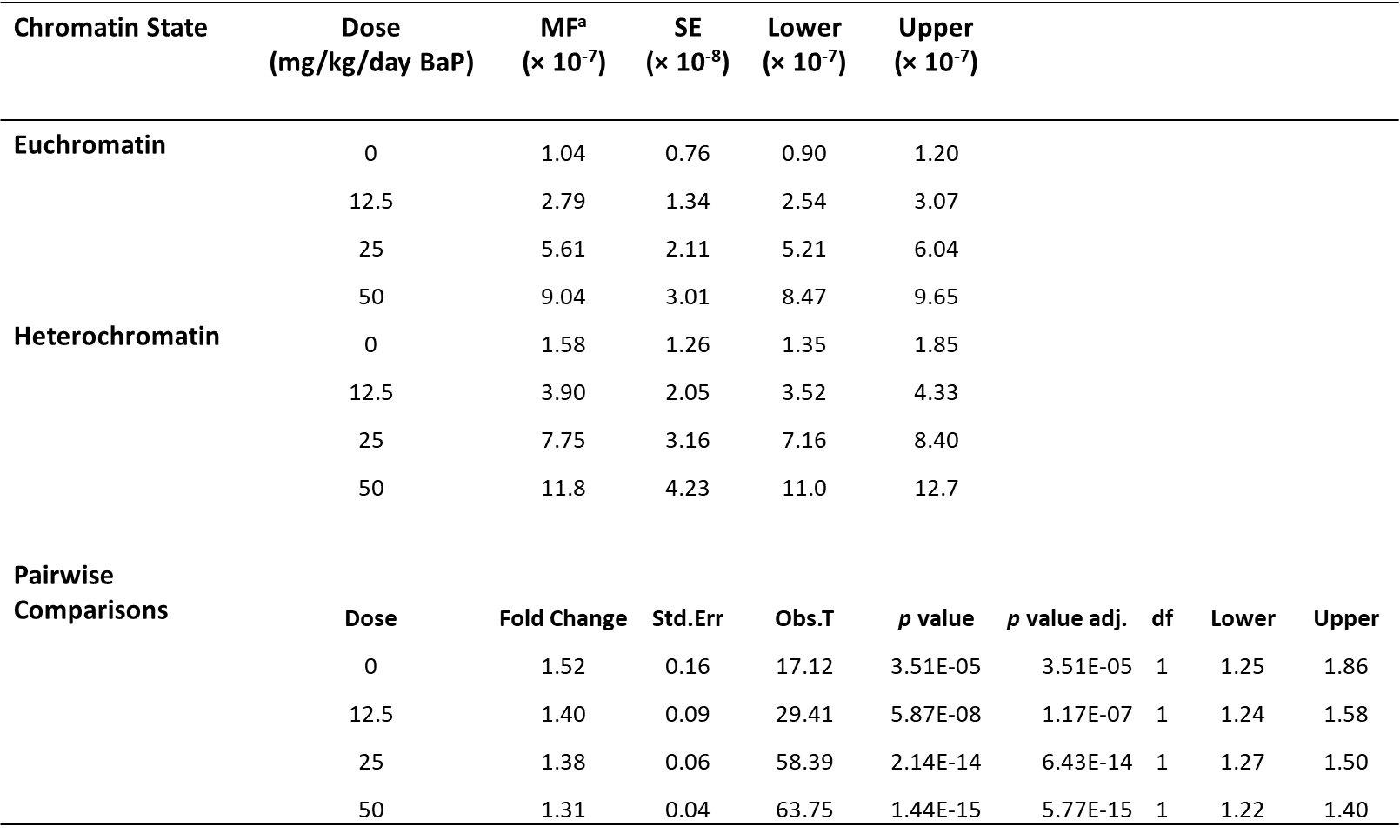
**

^a^DS MF shown as mutants per bp sequenced

Supplementary Figure 4. Proportion of CpG sites that were mutated in the bone marrow of MutaMouse animals. Data represent mean proportion of CpG sites across the mutagenesis panel that were mutated. Error bars represent SEM.

**
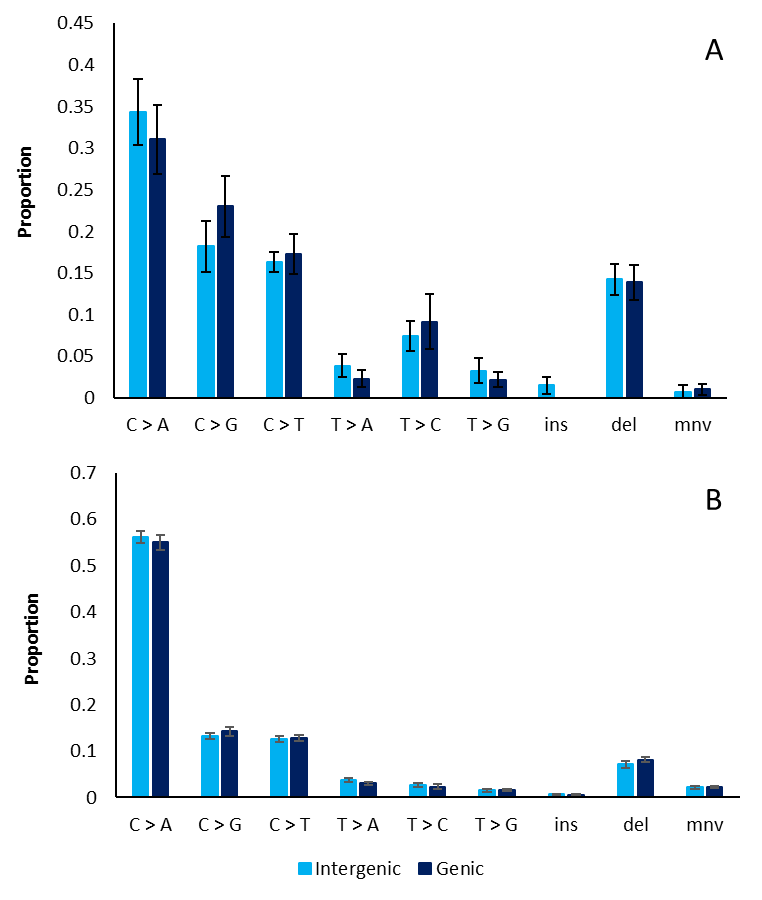
**

**Supplementary Figure 5. Proportion of mutation subtypes between intergenic and genic targets.** Mutation subtypes in controls (A) and BaP treated animals (B). MF are shown for intergenic (light blue) and genic (dark blue) targets. BaP-treated animal data is shown for all dose groups combined; however, the same pattern was observed with the dose groups considered separately.

**
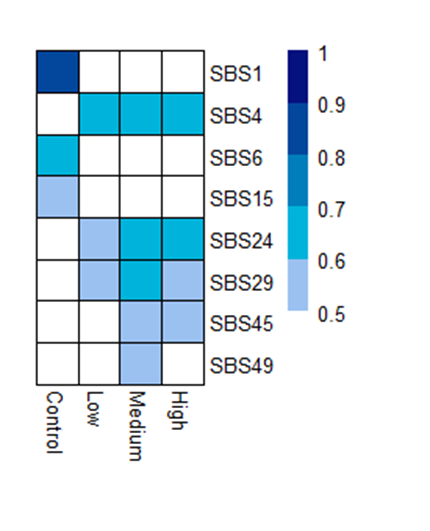
**

Supplementary Figure 6. Heatmap of cosine similarities for BaP induced mutational profiles and COSMIC Single Base Substitution signatures. Only cosine similarities that were equal to or above 0.5 are shown. Cosine similarities for all comparisons can be found in Supplemental Table 2. Note that SBS 4 is the COSMIC signature associated with tobacco smoke-induced lung cancer.


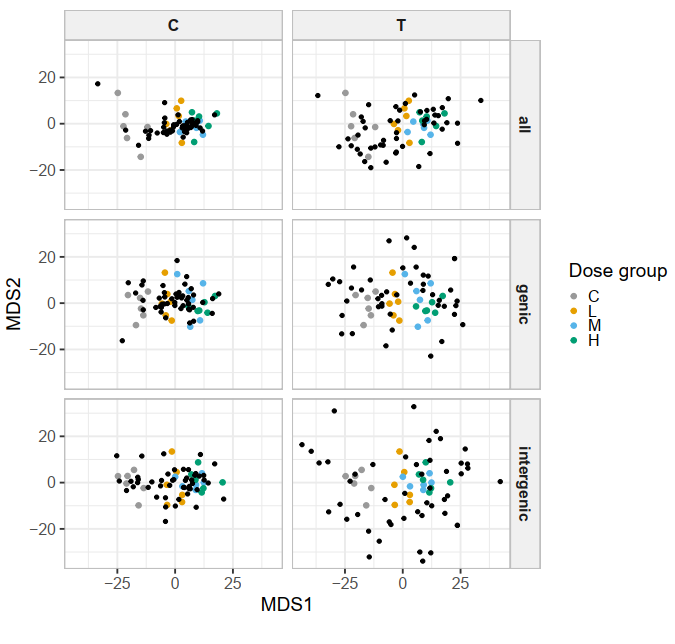


**Supplementary Figure 7**. **NMDS of binomial distances of trinucleotide mutation data, split by genomic regions (all, genic, intergenic).** Ordination for trinucleotide mutations is shown as black points, split by the reference base (C vs. T in the pyrimidine context). We observed that dose groups correlate with multidimensional scaling coordinate 1 (MDS1) and trinucleotide mutations with a T:A reference base are more dispersed and cluster further from BaP samples. Genic and intergenic binomial dissimilarity matrices were significantly correlated (Mantel statistic: 0.7478, significance: 0.001).


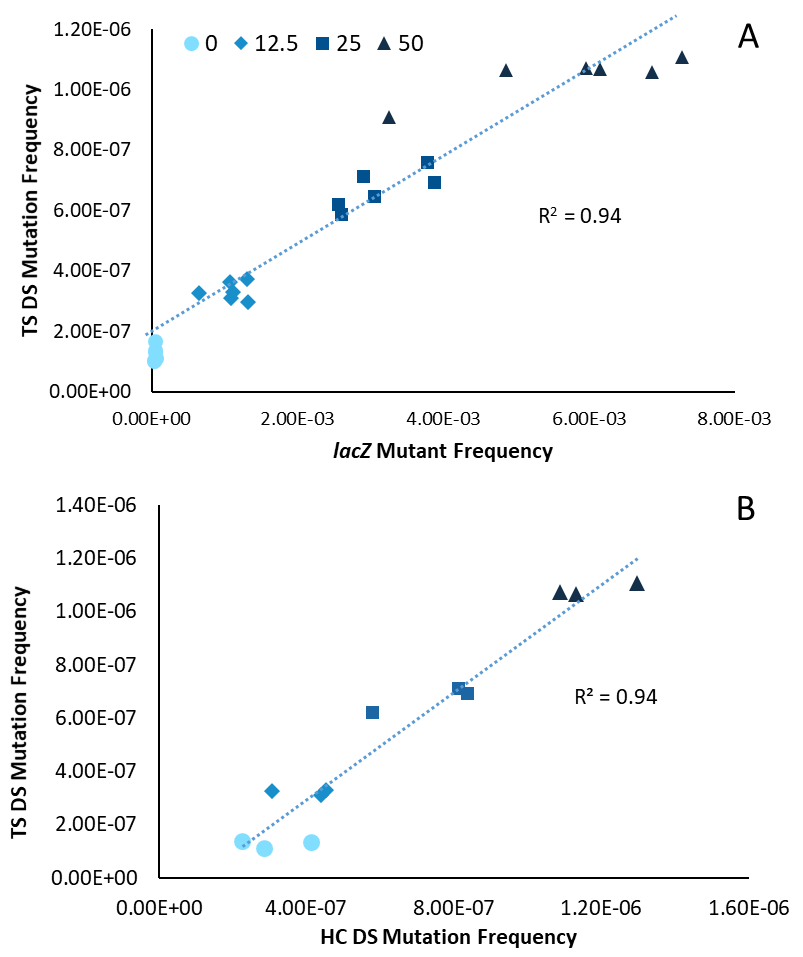


**Supplementary Figure 8. Correlation analysis of Duplex Sequencing mutation frequency (MF) and mutant frequency derived using the *lacZ* TGR assay (A) and of Duplex Sequencing MFs derived in an inter-laboratory validation between TwinStrand Biosciences (TS) and Health Canada (HC) (B).**

Data for mutation frequency correlation represented for each individual animal. Note that DS mutation frequencies are shown as mutants per bp sequenced and *lacZ* mutant frequencies are shown as mutants per locus.

**Supplementary Table 5. BaP induced insertion and deletion mutations in the bone marrow of MutaMouse animals.**

**
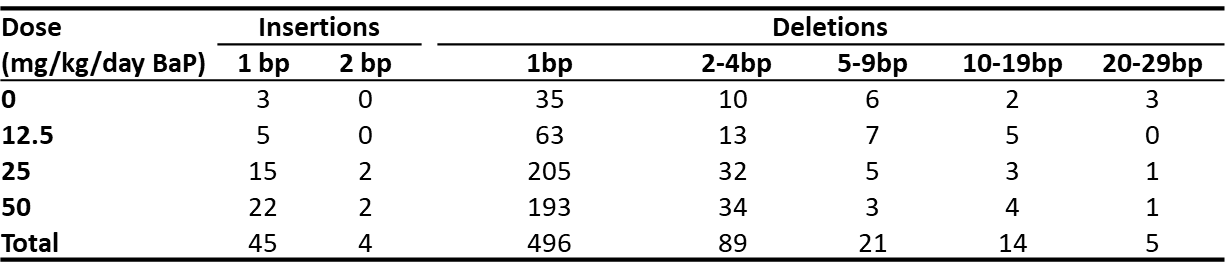
**

Table headings indicate length in base pairs (bp) of the insertion (ins) and deletion (del) mutations.

**R Script used to perform tertiary analysis for per-target mutation frequency calculation**

require(data.table)

require(assertthat)

require(dplyr)

require(binom)

require(GenomicRanges)

MUT_FILEPATH <- "all.mut"

VAF_CUTOFF <- c(0.3, 0.7, 0.85)

INTERVAL_FILE <- "all_intervals.bed"

OUT_FILEPATH <- "~/out.csv"

op <- list()

op$column.total_depth <- "total_depth"

op$column.n_depth <- "n_depth"

op$column.vaf <- "vaf"

op$column.alt_depth <- "alt_depth"

op$column.is.snp <- "is_snp"

op$column.mut_depth <- "mut_depth"

op$column.mut_freq <- "mut_freq"

op$column.chr <- "chr"

op$column.start <- "start"

op$column.end <- "end"

op$column.sample <- "sample"

op$column.lower_ci <- "lower_ci"

op$column.upper_ci <- "upper_ci"

op$site.columns <- c("contig", "start")

op$mut_count_method <- "min"

op$processed_required_mut_cols <-

c("mut_depth",

"total_depth",

"var_type",

"subtype",

"context",

"vaf")

op$base_required_mut_cols <-

c("chr",

"start",

"end",

"sample",

"ref",

"alt",

"alt_depth",

"depth",

"n_depth")

op$default_vaf_cutoffs <- c(0.3, 0.7, 0.9)

read_mut <- function (filepath, vaf_cutoffs = default_vaf_cutoffs) {

mut_preprocessing(migrate_mut(data.table::fread(filepath)),

vaf_cutoffs)

}

migrate_mut <- function (mut_table, processed = FALSE)

{

required_cols <- if (processed)

c(op$base_required_mut_cols, processed_required_mut_cols)

else

base_required_mut_cols

mut_table <- data.table::as.data.table(mut_table)

for (required_column in names(required_cols)) {

if (!op[[required_column]] %in% colnames(mut_table)) {

matching_column_indices <- which(colnames(mut_table) %in%

required_cols[[required_column]])

assertthat::assert_that(

length(matching_column_indices) ==

1,

msg = paste0(

"Found ",

length(matching_column_indices),

" columns matching ",

required_column,

":",

op[[required_column]],

". Instead of 1."

)

)

colnames(mut_table)[matching_column_indices] <-

op[[required_column]]

}

}

mut_table

}

mut_preprocessing <-

function (raw_mut_table,

vaf_cutoffs = op$default_vaf_cutoffs)

{

validate_mut_frame(raw_mut_table)

mut_table <-

raw_mut_table %>% mutate(`:=`(

!!sym(op$column.total_depth),!!sym(op$column.depth) -!!sym(op$column.n_depth)

))

mut_table <- mut_table %>% mutate(`:=`(

!!sym(op$column.vaf),!!sym(op$column.alt_depth) / !!sym(op$column.total_depth)

))

vaf_cutoffs <- sort(vaf_cutoffs)

mut_table <- mut_table %>% mutate(`:=`(

!!sym(op$column.is.snp),

(

!!sym(op$column.vaf) < vaf_cutoffs[2] & !!sym(op$column.vaf) >

vaf_cutoffs[1]

) | (!!sym(op$column.vaf) > vaf_cutoffs[3])

))

mut_table <-

mut_table %>% mutate(`:=`(

!!sym(op$column.is.snp.with.mut),

(

!!sym(op$column.vaf) > vaf_cutoffs[3] &

!!sym(op$column.total_depth) >

!!sym(op$column.alt_depth)

)

))

mut_table <-

mut_table %>% mutate(`:=`(!!sym(op$column.mut_depth),

ifelse(

!!sym(op$column.is.snp) & !(!!sym(op$column.is.snp.with.mut)),

0,

ifelse(

!!sym(op$column.is.snp.with.mut),!!sym(op$column.total_depth) -!!sym(op$column.alt_depth),!!sym(op$column.alt_depth)

)

)))

return(mut_table)

}

validate_mut_frame <- function (mut_df) {

required_cols <- op$base_required_mut_cols

all_required_cols_exist <- length(setdiff(required_cols,

colnames(mut_df))) == 0

if (!all_required_cols_exist) {

stop(paste("Missing required columns: ", paste(

setdiff(required_cols,

colnames(mut_df)), collapse = ","

)))

}

}

validate_processed_mut_frame <- function (mut_df)

{

required_cols <- op[names(processed_required_mut_cols)]

assertthat::assert_that(is.data.frame(mut_df))

validate_mut_frame(mut_df)

all_required_cols_exist <- length(setdiff(required_cols,

colnames(mut_df))) == 0

if (!all_required_cols_exist) {

stop(paste("Missing required columns: ", paste(

setdiff(required_cols,

colnames(mut_df)), collapse = ","

)))

}

}

grange_to_bed_df <- function (gr) {

op <- options()

if (!class(gr) == "GRanges") {

stop(paste0(

"Input should be a Grange object. Current input type: ",

class(gr),

"."

))

}

gr %>% as.data.frame() %>% select(-c("width", "strand")) %>%

rename(`:=`(!!sym(op$column.chr), "seqnames"),

`:=`(!!sym(op$column.start), "start"),

`:=`(!!sym(op$column.end),

"end")) %>% mutate(`:=`(!!sym(op$column.start),!!sym(op$column.start) - 1))

}

filter_mut_by_grange <- function (mut_df, region_grange)

{

op <- options()

validate_processed_mut_frame(mut_df)

if (nrow(mut_df) == 0) {

stop(paste0("Input mut_df should not be empty"))

}

if (!class(region_grange) == "GRanges") {

stop(paste0("Input data should be a GenomicRanges::GRange object"))

}

data_grange <- bed_df_to_grange(mut_df)

overlapping_data <-

grange_to_bed_df(IRanges::subsetByOverlaps(data_grange,

region_grange))

overlapping_data[, op$column.chr] <-

as.character(overlapping_data[,

op$column.chr])

overlapping_data

}

calculate_mut_freq <-

function (mut_df,

unique_sample_columns = op$column.sample,

calculate_mutfreq_by = op$column.sample,

method = op$mut_count_method,

max_vaf = 0.01) {

required_columns <- c(unique_sample_columns, calculate_mutfreq_by)

assertthat::assert_that(

all(required_columns %in% colnames(mut_df)),

msg = paste0(

"\"",

paste(required_columns[which(!required_columns %in%

colnames(mut_df))], collapse = ", "),

"\" not included in input dataframe"

)

)

assertthat::assert_that(

nrow(count(

mut_df,!!!sapply(unique(unique_sample_columns),

sym, USE.NAMES = FALSE)

)) == length(unique(mut_df[[op$column.sample]])),

msg = paste(

"Together, the values in the",

paste(unique_sample_columns,

collapse = ", "),

"column(s) must generate as many unique combinations as there are unique values in the",

op$column.sample,

"column."

)

)

unique_group_by_columns <- unique(c(

unique_sample_columns,

op$site.columns,

calculate_mutfreq_by

))

summarize_by_columns <- unique(calculate_mutfreq_by)

summarized_data <- left_join(

calculate_total_depths(mut_df,

unique_group_by_columns, summarize_by_columns),

calculate_mutation_counts(mut_df,

summarize_by_columns, max_vaf, method),

by = calculate_mutfreq_by

)

colnames(summarized_data)[(ncol(summarized_data) - 1):ncol(summarized_data)] <-

c(op$column.total_depth,

op$column.mut_depth)

summarized_data_with_wilson <-

add_binom_conf_intervals(summarized_data,

op$column.mut_depth,

op$column.total_depth,

method = "wilson")

colnames(summarized_data_with_wilson)[ncol(summarized_data_with_wilson) - 2] <-

op$column.mut_freq

summarized_data_with_wilson

}

calculate_total_depths <-

function (mut_df,

unique_group_by_columns,

summarize_by_columns)

{

mut_df %>% group_by_at(unique_group_by_columns) %>% summarise(total_bases = first(.data[[op$column.total_depth]]),

.groups = "keep") %>% ungroup() %>% group_by_at(summarize_by_columns) %>%

summarise(total_bases = sum(.data[["total_bases"]])) %>%

ungroup()

}

calculate_mutation_counts <-

function (mut_df,

summarize_by_columns,

max_vaf,

method)

{

vaf_sym <- sym(op$column.vaf)

group_by_at(filter(mut_df, 0 < !!vaf_sym,!!vaf_sym <= max_vaf,!(!!sym(op$column.is.snp))),

summarize_by_columns) %>%

summarise(mut_count = ifelse(method == "min", length(.data[[op$column.alt_depth]]),

sum(.data[[op$column.alt_depth]])))

}

add_binom_conf_intervals <-

function (df,

x,

n,

conf.level = 0.95,

method = "wilson")

{

if (length(method) != 1 || method == "all") {

stop("Must select only one method.")

}

df <- as.data.frame(df)

not_included <- setdiff(c(x, n), colnames(df))

if (length(not_included) > 0) {

stop(paste0(

"Input dataframe does not include all required columns: ",

paste(not_included, collapse = ", ")

))

}

if (!is.numeric(df[[x]]) | !is.numeric(df[[n]])) {

stop(paste0(

"x (",

x,

", ",

class(df[[x]]),

") and n (",

n,

", ",

class(df[[n]]),

") must be numeric."

))

}

if (nrow(df) == 0) {

df_ci <- data.frame(numeric(0), numeric(0), numeric(0))

colnames(df_ci) <- c("mean", op$column.lower_ci,

op$column.upper_ci)

}

else {

df_ci <- bind_rows(mapply(function(x_val, n_val) {

if (is.na(x_val) || is.na(n_val)) {

data.frame(

method = NA_character_,

x = NA_integer_,

n = NA_integer_,

mean = NA_integer_,

lower = NA_integer_,

upper = NA_integer_

)

}

else {

binom::binom.confint(x_val,

n_val,

conf.level = conf.level,

method = method)

}

}, df[, x], df[, n], SIMPLIFY = FALSE)) %>% select("mean",

"lower", "upper") %>% rename(`:=`(!!op$column.lower_ci,

"lower"),

`:=`(!!op$column.upper_ci,

"upper"))

}

cbind(df, df_ci)

}

bed_df_to_grange <- function (bed_df, flanking = 0) {

op <- options()

assertthat::assert_that(is.data.frame(bed_df))

assertthat::assert_that(flanking >= 0, msg = "Flanking region must be >= 0")

if (nrow(bed_df) == 0) {

stop(paste0("Input dataframe should have at least one row."))

}

else {

colnames(bed_df)[c(1:3)] <- c(op$column.chr, op$column.start,

op$column.end)

bed_df_for_gr <- bed_df %>% mutate(`:=`(!!sym(op$column.start),!!sym(op$column.start) + 1)) %>% mutate(`:=`(!!sym(op$column.start),

pmax(1,!!sym(

op$column.start

) - flanking)),

`:=`(!!sym(op$column.end),!!sym(op$column.end) +

flanking))

GenomicRanges::makeGRangesFromDataFrame(

bed_df_for_gr,

keep.extra.columns = TRUE,

ignore.strand = TRUE,

seqnames.field = op$column.chr

)

}

}

mut_data <-

read_mut(file.path(MUT_FILEPATH), vaf_cutoffs = op$default_vaf_cutoffs)

sample_mut_freq <- calculate_mut_freq(

mut_data,

calculate_mutfreq_by = "sample",

method = "min",

max_vaf = 0.01

)

target_df <- as.data.frame(data.table::fread(INTERVAL_FILE))

sample_mf_by_roi <- lapply(all_region_names, function(x) {

mut_roi <-

filter_mut_by_grange(mut_data, bed_df_to_grange(target_df %>% filter(V4 ==

x)))

sample_mut_freq <-

calculate_mut_freq(

mut_roi,

calculate_mutfreq_by = "sample",

method = "min",

max_vaf = 0.01

)

}) %>% bind_rows(.)

write.csv(sample_mf_by_roi, file = OUT_FILEPATH)
